# Relationship between reasons for intermittent missing patient-reported outcomes data and missing data mechanisms

**DOI:** 10.1007/s11136-024-03707-y

**Published:** 2024-06-16

**Authors:** Lene Kongsgaard Nielsen, Rebecca Mercieca-Bebber, Sören Möller, Louise Redder, Mary Jarden, Christen Lykkegaard Andersen, Henrik Frederiksen, Asta Svirskaite, Trine Silkjær, Morten Saaby Steffensen, Per Trøllund Pedersen, Maja Hinge, Mikael Frederiksen, Bo Amdi Jensen, Carsten Helleberg, Anne Kærsgaard Mylin, Niels Abildgaard, Madeleine T. King

**Affiliations:** 1 Department of Haematology, Quality of Life Research Center, Odense University Hospital, Odense, Denmark; 2 Section of Haematology, Department of Internal Medicine, Gødstrup Hospital, Herning, Denmark; 3 University of Sydney, NHMRC Clinical Trials Centre, Sydney, NSW Australia; 4 OPEN, Open Patient Data Explorative Network, Odense University Hospital, Odense, Denmark; 5 Department of Clinical Research, University of Southern Denmark, Odense, Denmark; 6 Department of Haematology, Copenhagen University Hospital, Copenhagen, Denmark; 7 Department of Clinical Medicine, Faculty of Health and Medical Sciences, University of Copenhagen, Copenhagen, Denmark; 8 Department of Public Health, Research Unit for General Practice and Section of General Practice, University of Copenhagen, Copenhagen, Denmark; 9 Department of Haematology, Aalborg University Hospital, Aalborg, Denmark; 10 Department of Haematology, Aarhus University Hospital, Aarhus, Denmark; 11 Department of Haematology, South West Jutland Hospital, Esbjerg, Denmark; 12 Department of Haematology, Vejle Hospital, Vejle, Denmark; 13 Department of Haematology, Hospital of Southern Jutland, Aabenraa, Denmark; 14 Department of Haematology, Zealand University Hospital, Roskilde, Denmark; 15 School of Psychology, University of Sydney, Sydney, Australia

**Keywords:** Patient-reported outcomes, Missing data, Missing data mechanism, Multiple myeloma, Quality of life

## Abstract

**Purpose:**

Non-response (NR) to patient-reported outcome (PRO) questionnaires may cause bias if not handled appropriately. Collecting reasons for NR is recommended, but how reasons for NR are related to missing data mechanisms remains unexplored. We aimed to explore this relationship for intermittent NRs.

**Methods:**

Patients with multiple myeloma completed validated PRO questionnaires at enrolment and 12 follow-up time-points. NR was defined as non-completion of a follow-up assessment within seven days, which triggered contact with the patient, recording the reason for missingness and an invitation to complete the questionnaire (denoted “salvage response”). Mean differences between salvage and previous on-time scores were estimated for groups defined by reasons for NR using linear regression with clustered standard errors. Statistically significant mean differences larger than minimal important difference thresholds were interpreted as “missing not at random” (MNAR) mechanism (i.e. assumed to be related to declining health), and the remainder interpreted as aligned with “missing completely at random” (MCAR) mechanism (i.e. assumed unrelated to changes in health).

**Results:**

Most (7228/7534 (96%)) follow-up questionnaires were completed; 11% (802/7534) were salvage responses. Mean salvage scores were compared to previous on-time scores by reason: those due to hospital admission, mental or physical reasons were worse in 10/22 PRO domains; those due to technical difficulties/procedural errors were no different in 21/22 PRO domains; and those due to overlooked/forgotten or other/unspecified reasons were no different in any domains.

**Conclusion:**

Intermittent NRs due to hospital admission, mental or physical reasons were aligned with MNAR mechanism for nearly half of PRO domains, while intermittent NRs due to technical difficulties/procedural errors or other/unspecified reasons generally were aligned with MCAR mechanism.

**Supplementary Information:**

The online version contains supplementary material available at 10.1007/s11136-024-03707-y.

## Plain English summary

Missed completion of questionnaires in quality of life studies can be a problem for how the results of the questionnaire are analyzed and interpreted. This is because if participants with poor quality of life do not complete the questionnaire, the analyzed data may not be representative of the health of the entire sample. In this project we investigated whether there is a relationship between the participants´ quality of life when they failed to complete a questionnaire and participants’ reason for being unable to missed complete the questionnaire, in a registry-based survey of people with multiple myeloma. Our findings suggest that if participants do not complete quality of life questionnaires due to poor health (as indicated by a hospital admission, physical or mental reasons), there is a high risk that the participants experience poor quality of life at the time. For analysis purposes, we refer to this as “missing not at random” or “MNAR” mechanism. This relationship was demonstrated for 10/22 health domains tested. However, if the participants reported that the reason for missed completion was an oversight, their quality of life was, on average, no different to their previous scores, and we can consider this “missing completely at random” or MCAR mechanism (for 21/22 domains tested). Knowing this reinforces the need to collect reasons for missing data and allows researchers to understand how much bias different types of missing data will cause when analyzing and interpreting questionnaire results from clinical trials or registries.

## Background

Patients’ experience of symptoms of disease, side effects of treatment and impact on quality of life (QoL) can be validly and reliably captured with patient-reported outcome (PRO) questionnaires [1, 2]. However, non-response (NR) to PRO questionnaires can lead to loss of study power and precision, and difficulties in data interpretation [3, 4]. NRs can be categorized according to the patterns of missingness; (1) Monotone missingness occurs when scheduled questionnaires are completed until the patient drops out of the study. (2) Intermittent missingness occurs if one or more scheduled questionnaires are not completed between completed questionnaires. (3) Mixed missingness is used if intermittent missingness occurs initially, and then the patient drops out [4, 5].

NR leads to missing data, requiring careful consideration during data analysis and interpretation. All methods for analyzing longitudinal data with missing data make assumptions about the underlying mechanisms of the any missing data [6]. Statisticians define three categories of missing data mechanisms [6]: “*Missing completely at random*” (MCAR), which is when the probability of missingness is unrelated to the outcome and unobserved PRO score, and unrelated to observed PRO data. MCAR data occur, for example, if a staff member forgets to provide the questionnaire to the patient. “*Missing at random*” (MAR) is when the probability of missingness may be related to observed PRO data or covariates, but not to the unobserved PRO score. An example of MAR could be a higher proportion of missing data from older participants, who tend to have worse PRO scores [7]. “*Missing not at random*” (MNAR) is when the probability of missingness is related to the unobserved PRO score. MNAR data occur if PRO assessments are likely to be missed when patients’ health (related to measured patient-reported outcomes) is declining, and/or they are experiencing adverse events or complications. MNAR data are therefore termed ‘non-ignorable’, as not addressing such missing data is likely to lead to bias [4, 8]. To ensure that missing data within a dataset are handled appropriately, ideally additional data or auxiliary variables should be collected to inform researchers about the missing data mechanisms as this can inform appropriate statistical handling of the missing data [9]. This is important, since often the missing data mechanism has stronger impact on the results than the proportion of missing data does [3, 7]. However, currently there are no straightforward methods for determining missing data mechanisms, particularly in datasets with multiple missing observations involving more than one missing data mechanism [10]. Although collecting reasons for NR is a recommended management strategy, the empirical relationship between common reasons for NR and missing data mechanisms has not yet been investigated.

Multiple myeloma (MM) is an incurable malignancy of the plasma cells in the bone marrow associated with severe morbidity caused by painful bone destruction, bone fractures, bone marrow failure, high infection rates, renal dysfunction, and physical disability [11, 12]. Treatment for MM typically involves repeated cycles of combined cyto-reductive therapy with the risk of acute adverse events for the patients, e.g. infections leading to hospital admissions, and late effects, e.g., peripheral neuropathy, pain and fatigue [13–17]. Evidence-based knowledge of health-related QoL (HRQL) is critical in developing new treatments, shared decision making and for improvement of supportive care [1]. A review of methodological aspects of HRQL assessment in published longitudinal studies of patients with MM found an intermittent NR rate of 22%, and rates between 28 and 96% for monotone NR [18]. Missing data mechanisms were investigated or sensitivity analyses presented, in only 8 out of 23 studies. These findings suggest that the current knowledge of HRQL in MM is compromised by NR, affecting the generalizability of findings and their applicability to daily clinical practice [18].

To establish reliable evidence-based knowledge of HRQL for patients with MM and other patient groups where missing PRO data are a consequence of the disease or its treatment, recommendations for appropriate handling of missing data are needed. Further, appropriate handling of missing data will vary according to the underlying missing data mechanism (MNAR, MAR, MCAR) [10]. In order to determine the missing data mechanism(s) at play in any particular data set originating from a survey, clinical trial or cross-sectional study, it would be helpful to understand the relationship between commonly reported reasons for intermittent NRs and the missing data mechanisms defined probabilistically by statisticians. Our aim was to explore the relationship between reasons for intermittent NRs and underlying missing data mechanisms in the cohort of patients with multiple myeloma included in the study of “Quality of life in Danish multiple myeloma patients”(QoL-MM).

## Methods

### Design and patients

This study utilized the patient cohort included in QoL-MM, a prospective, multicenter, observational and primarily web-based survey with real-time monitoring of NRs. PRO study design, data collection procedures and strategies to minimize intermittent NR have been published previously [19]. In brief, newly diagnosed or relapsed patients with MM were eligible for participation in the study at the time of starting treatment. Patients were excluded if they did not understand the Danish language or had a psychiatric diagnosis or mental illness that prevented answering the questions in the questionnaires. The study was closed in November 2023, at which time all enrolled participants had completed the study, including 2-year follow-up.

### PRO data collection

Patients completed validated questionnaires, chosen for their relevance to patients with MM and MM treatment, at recruitment and during 12 follow-up time points over 2 years. The cancer-specific HRQL questionnaire of the European Organisation for Research and Treatment of Cancer (EORTC) Quality of life—Core 30 (EORTC QLQ-C30) and the Chemotherapy-Induced Peripheral Neuropathy module (EORTC QLQ-CIPN20) were provided every 4 weeks for the initial 6 months and thereafter every 3 months, until 2 years. The Multiple Myeloma module QLQ-MY20 (EORTC QLQ-MY20) and the Short-form health survey, version 2 (SF12v2) were included at 3, 6, 12, 18 and 24 months.

- EORTC QLQ-C30 contains 30 items that summarize as five functional domains (physical, role, emotional, cognitive and social), eight symptom domains/items (fatigue, nausea and vomiting, pain, dyspnoea, insomnia, appetite loss, constipation and diarrhea), and an item about financial difficulties and one global health status/QoL scale [20].
- EORTC QLQ-MY20 contains 20 items that summarize as two functional domains (future perspectives and body image) and two symptom domains (disease symptoms and side effects of treatment) [21].
- EORTC QLQ-CIPN20 contains 20 items that summarize as an 18-item peripheral neuropathy sum score, which is a multi-item symptom domain excluding items 19 and 20 [22].
- SF12v2 contains 12 items that summarize as two component summary scores (physical and mental) [23].

The EORTC questionnaires have a recall period of 7 days, whereas the SF12v2 questionnaire has a 4-week recall period. For all four questionnaires, higher scores indicate better functioning for the functional domains and component summary scores, whereas they indicate more severe symptoms for the symptom domains.

### Collection of reason for non-responses

Real-time, central monitoring of intermittent NRs for both web-based and paper-based PRO questionnaire completion was carried out [19]. An automatic reminder was sent to each patient who had not completed an electronic questionnaire by day 4 after the scheduled day for completion (target date). A response to a follow-up PRO assessment was defined as completion of at least one item in the EORTC QLQ-C30 or the EORTC QLQ-CIPN20 questionnaires. If a patient had not answered any of the items in the EORTC QLQ-C30 or the EORTC QLQ-CIPN20 within 7 days from the target date, that questionnaire was defined as NR. Every weekday (Monday to Friday), the central study office monitored for NR, and in case of NR, the study office sent an email notification to the local study nurse. Within a 2-weekday timeframe starting at the end of the 7-day window, the local nurse contacted the patient to determine and document the reason for NR from a pre-specified list of 11 reasons for non-response, presented in Table 1. The local nurse then invited the patient to complete the questionnaire, using the recall periods specified in the questionnaires and not the time the assessment was initially scheduled. If the local study nurse was not able to get in contact with the patient within the 2-weekday timeframe, the local study nurse used the pre-specified reason “*9. Not possible to get in contact with the patient and identify the reason*”. In such cases, the local study nurse was instructed to meet the patient at the next visit in the clinic to clarify the reason by asking the patient directly and revise the documented reason correspondingly. A detailed description of the procedures for ascertaining reasons for missing data can be found in the supplementary appendix.

**Table 1:** List of pre-specified reasons for intermittent non-responses, grouping of reasons for non-response and the expected missing data mechanism

Reasons for intermittent non-responses	Expected missing data mechanism
*Non-responses group 1*	
The patient is admitted to the Hematological Department	Missing not at random
The patient is admitted to another department than the Hematological Department	
The patient was not physically capable of answering the questionnaire	
The patient was not mentally capable of answering the questionnaire	
*Non-responses group 2*	
The patient had technical difficulties in answering the questionnaire	Missing completely at random
The patient has never received the questionnaire (electronic or paper)	
The paper questionnaire has disappeared and cannot be uploaded	
*Non-responses group 3*	
The patient has overlooked/forgotten the questionnaire (electronic or paper)	Missing completely at random
*Non-responses group 4*	
Not possible to get in contact with the patient and find the reason	Uncertain
Other reason for not answering the questionnaire	
*Recoded*	
The patient is deceased (Recoded to monotone missing data, and hence excluded from this substudy)	

Questionnaires completed within the 7-day window were categorized as “on-time responses”. When a patient completed the questionnaire after the 7-day window, the response was referred to as a “salvage response” aligned with a reminder response. If the patient did not complete the questionnaire, it was categorized as a “never response”. No predefined end date for questionnaire completion was included in the study design, however overlapping time windows were not a challenge, as only 4% (48/1367) were completed after day 9, and very few after day 15 [19]. All data were collected and stored in a REDCap database [24]. A detailed description of real-time monitoring can be found in the supplementary appendix.

Early patient drop-out and termination of PRO data collection were defined as instances where the patient withdrew consent, entered a permanent state of inability to provide answers to the questionnaires (e.g., dementia) or if the patient died. These represent monotone (terminal) missing data and were not considered in this paper. As a result of real-time monitoring, the reason for every salvage and never response was collected. Intermittent NRs were defined as scheduled follow-up assessment not completed within the 7-day window with exception of monotone non-responses. There were two types of intermittent non-responses: salvage responses (completion later than seven days after the target day following the reminder) and never responses (non-completion of a scheduled follow-up assessment at any time).

## List of key terms and definitions

**Table:** 

Non-response	Non-completion of a scheduled follow-up assessment (any items of EORTC QLQ-C30 or EORTC QLQ-CIPN20) within seven days from the target day
Intermittent non-response	Non-completion of a scheduled follow-up assessment (any items of EORTC QLQ-C30 or EORTC QLQ-CIPN20) within seven days from the target day with exception of monotone non-responses. There are two types of intermittent non-responses: salvage responses and never responses
Salvage response	Completion of a scheduled follow-up assessment later than seven days after the target day
Never response	Non-completion of a scheduled follow-up assessment at any time
On-time response	Completion of a scheduled follow-up assessment within the seven day time window
Previous on-time score before salvage response	The last on-time PRO response score prior to a salvaged response score. If the PRO score before the salvage response was missing or salvaged, the most recent on-time response score before that one was defined as the previous on-time score
Previous on-time score before never response	The last on-time PRO scores prior to a never response. If the PRO score before the never response was missing or salvaged, the most recent on-time response score before that one was defined as the previous on-time score before the never response
Monotone non-response	Completion of scheduled follow-up assessments until the time of drop out and no further assessments are completed (not considered in this paper)

*EORTC QLQ-C30* European Organisation for Research and Treatment of Cancer Quality of life—Core 30; *EORTC QLQ-CIPN20* European Organisation for Research and Treatment of Cancer Chemotherapy-Induced Peripheral Neuropathy module; *PRO* patient-reported outcome

### Data analysis and interpretation

The analyzed patient cohort was all patients included in the QoL-MM survey. The analyzed questionnaires were all follow-up assessments from the analyzed patient cohort starting at 4-weeks follow-up until 24 months follow-up or drop-out for any reason (which ever came first). We corrected for dependence among observations from the same patient by using mixed models with clustered standard errors estimated with robust sandwich estimators.

The pre-specified reasons for intermittent NR were grouped a priori into four NR groups based on the assumption of having the same underlying missing data mechanisms; *NR group 1*: NR due to hospital admission, physical or mental reasons; *NR group 2:* NR due to technical difficulties, never received questionnaire, or paper disappeared; *NR group 3:* NR due to patients forgetting to complete or overlooking the questionnaires; and *NR group 4:* NR due to other or no available reason for NR. The four NR groups are presented in Table 1. NR group 1 was divided into two subgroups: *NR group 1a*: NR due to hospital admission and *NR group 1b*: NR due to physical or mental reasons. We expected that NR due to hospital admission or mental or physical reasons (NR group 1) would align with the MNAR mechanism, i.e., it was assumed these patients were experiencing poorer outcomes at this time-point than at their previous on-time assessment. For the remaining three NR groups (NR group 2–4), we expected that NR would align with the MCAR mechanism, i.e., the outcomes experienced at these time points were similar to those experienced at the time of previous on-time assessment [4].

The PRO domain scores were calculated for each patient at each scheduled HRQL assessment time point using the related scoring manuals [23, 25]. All “never responses” were set to “missing”. The remainder were identified as either “on-time” or “salvage responses”. As the domain scores for never responses remained missing, the analyses of intermittent NRs could only be conducted on the salvage responses, using the last previous on-time score before salvage response for comparison. Therefore, for each salvage response, the last previous on-time score was used, even if there were two or more adjacent salvage responses. Thus, it was expected that for NR Group 1, the mean salvage score would represent poorer health outcomes (i.e., lower mean scores for the functional domains, higher mean scores for symptom domains) than the mean previous on-time score before salvage response. For the remaining three NR groups, it was expected that the mean salvage score would not differ from the mean previous on-time score before salvage response.

For each NR group, the mean salvage response score was compared to the previous on-time score before salvage response by linear regression with clustered standard errors, taking into account the fact that the same patient might contribute to more than one pair of data points (salvage, previously on-time score) and appear in more than one group. Mean differences and standard differences are presented together with 95% confidence intervals. As not all patients provided salvage responses, a post hoc analysis was performed, where the mean previous on-time score before salvage responses was compared to mean previous on-time score before never responses.

Differences with p-values < 0.05 were considered statistically significantly different from zero; as this was an explorative study, we did not perform multiple testing correction. For statistically significant differences, we also assessed clinical relevance, defined as a minimal important difference (MID) of > 0.3 standard deviations (SD) of the baseline mean score for the entire group. Justification for this choice of MID was as follows. First, the effect size approach to interpreting PRO results is useful when analyzing a number of questionnaires (4 in this study), each of which yields a number of domain scales (22 in this study), because it standardizes interpretation across all these scales [26, 27]. Next, to determine the effect size threshold relevant for myeloma patients, we drew on evidence from the following three papers. Kvam et al. [28] investigated MIDs for QLQ-C30 global QoL, physical functioning, fatigue and pain for MM patients, using two anchor-based methods, and they that found effect sizes in the range 0.3SD to 0.5SD were appropriate. However, as this referred to only four of the 22 PRO domains of interest in this study, we looked to the broader evidence about effect sizes for PROs, and found that a medium effect size of 0.5SD has been found to be larger than the MID for cancer patients [29, 30]. Therefore, we settled on a threshold of 0.3 standard deviations of the baseline mean score of our study sample (all patients) as our MID. (For interested readers, Table S9 in the supplementary appendix shows that our 0.3SD MID threshold, when expressed as a raw score based on published baseline standard deviations, generally falls within the size range considered ‘small but clinically important’, which is very similar to the concept of minimally important difference). A statistically significant mean score difference that exceeded (worse or better) the MID was considered evidence for the missing data mechanism of MNAR. The remainder was considered evidence for missing data mechanism of MCAR. Baseline characteristics and the proportions of salvage responses of each NR group were analyzed using descriptive statistics. As the number of completed questionnaires varied slightly by PRO domain due to missing items, the number of available scores for the physical functioning domain is stated in the text, tables and figures, unless otherwise stated. All statistical analyses were performed using Stata 18.

## Results

681 patients were included in QoL-MM. In total, we expected 7534 scheduled follow-up questionnaires from the 681 patients. Mean age at entry was 68.4 years (standard deviation 9.2), more males (59%) than females were included, and 59% of the patients were included at the time of starting first-line treatment for MM. Electronic completion of the follow-up questionnaires was chosen by 84% of the patients. Patient characteristics are presented in the supplementary appendix Table S1.

The patients completed 7228 of the 7534 (96%) scheduled follow-up questionnaires. Most completions were on-time (6426/7534 (85%)), and 802/7534 (11%) were salvage responses, with 306/7534 (4%) never responses. The number of patients with complete data was 505, defined as patients without never responses. There was a higher rate of NR for paper completion (258/1082, 24%) compared to electronic completion (850/6452, 13%). The main reason for NR was overlooked/forgotten questionnaires (576/1108, 52%). The number of NRs due to hospital admission, physical or mental reasons were 286 out of 1,108 (26%). Overall, the proportion of salvage responses obtained after real-time monitoring was 72% (802/1108), with the highest proportion (91%, 523/576, p-value < 0.001) achieved in NR group 3 of forgotten/overlooked questionnaires (Table 2). Reasons for NR, salvage and never responses, divided into the pre-specified reasons for all NRs, electronic and paper completion are presented in the supplementary appendix, Tables S2–S4 and Figure S1. An addition, the number of patients with NRs, who had reported reasons for NR in NR patient group 1, 2, 3, 4 and mixed NR patient groups is presented in the supplementary appendix, Table S5.

**Table 2:** Reasons for non-responses, number of salvage and never responses divided into the four non-responses groups

	Number (%) of times reason cited for non-response	Number of salvage responses^a^	Proportion of non-responses salvaged^a^ (%)	Number of never responses
Non-responses	1108	802	72	306
Non-responses group 1 Due to hospital admission, physical or mental reasons	286 (26%)	113	40	173
Non-responses group 2 Due to technical difficulties, never received the questionnaire or paper disappeared	139 (13%)	111	80	28
Non-responses group 3 Due to overlooked/forgotten questionnaire	576 (52%)	523	91	53
Non-responses group 4 Due to other or no available reason for non-responses	107 (10%)	55	51	52

^a^Proportion of non-responses completed after the 7-day window following contact from study nurse

### Relationship between reasons for non-responses and missing data mechanisms

**Fig. 1:**
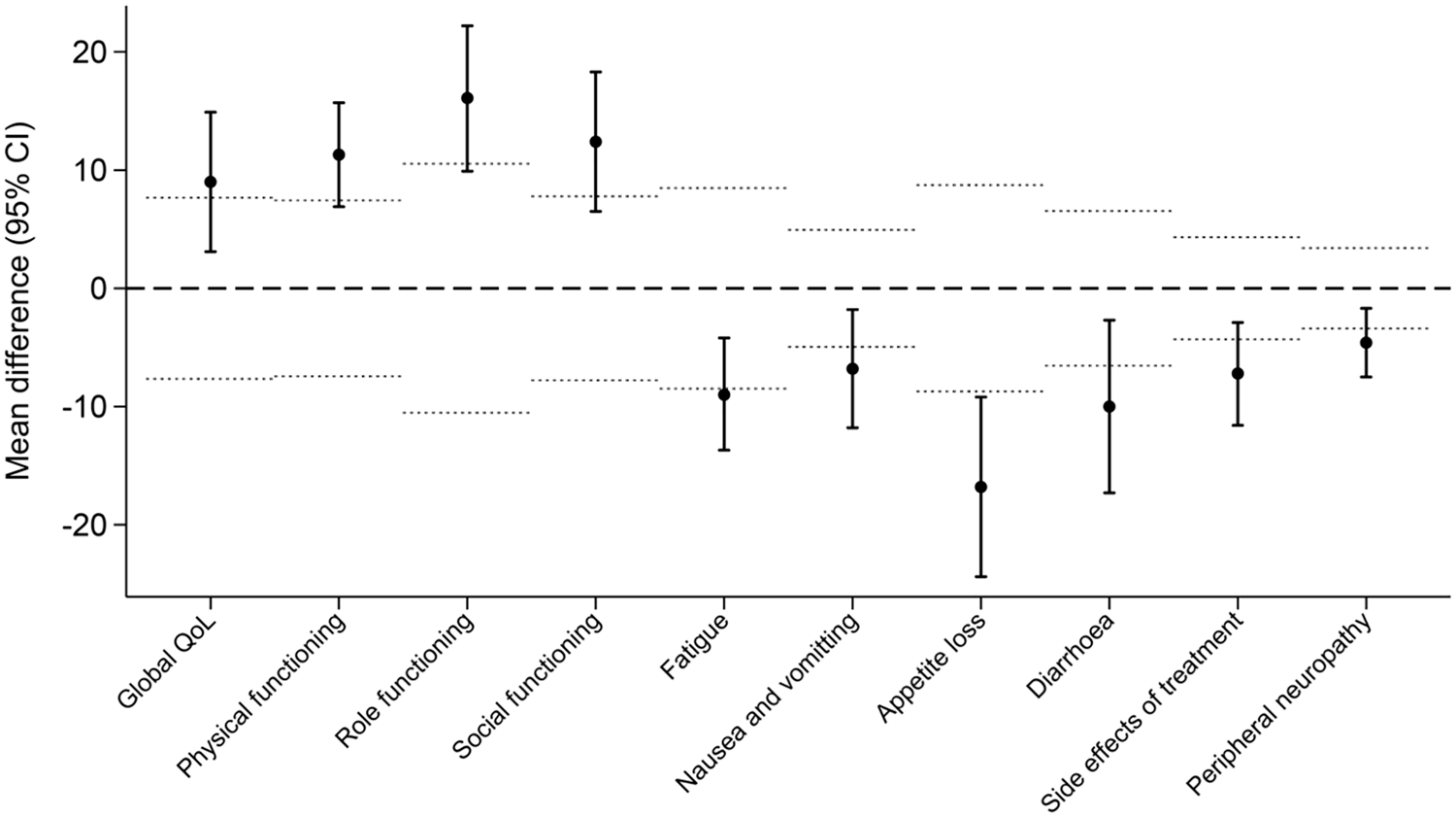
For 10 out of 22 domains of non-responses group 1, the mean salvage responses were statistical significant and clinically different compared to the mean previous on-time score before salvage responses. The dots are the mean score differences, the vertical bars represents 95% confidence intervals (CI) and the horizontal dotted lines represent the upper and lower minimal important difference for the individual domain. *QoL* quality of life

Considering subgroups by reasons for NR, the mean salvage scores for NR group 1 (due to hospital admission, physical or mental reasons) were statistically significantly and clinically worse in 10 out of 22 domains compared to the previous on-time mean scores before salvage response. This finding was therefore considered MNAR of the NRs for the 10 domains of global QoL, physical, role and social functioning, fatigue, nausea and vomiting, appetite loss, diarrhea, side effects of treatment and peripheral neuropathy. The mean scores, mean difference and p-values for NR group 1 are presented in Table 3 and Fig. 1. The subgroup analysis of NR group 1a (due to hospital admission) and NR group 1b (due to physical or mental reasons) revealed that, for 12 and four domains, respectively, the mean salvage score was statistically significant and clinically worse than the previous on-time score suggesting that NR due to hospital admission in particular, is aligned with MNAR. The mean scores, mean difference and p-values for NR group 1a and 1b are presented in Table S6 and S7 in the supplementary appendix.

**Table 3:** Mean differences non-responses group 1

Quality of life domains (minimal important difference threshold)	Mean previous on-time score before salvage response (SD)	Mean salvage score (SD)	Mean difference (95% Confidence interval)	Standardized difference (95% confidence interval)	p-value
*EORTC QLQ-C30*	Q = 113	Q = 113			
Global quality of life (7.66)	53.7 (25.3)	44.7 (24.3)	9.0 (3.1; 14.9)	0.4 (0.1; 0.6)	**0.003**
Physical functioning (7.44)	68.1 (22.9)	56.8 (23.9)	11.3 (6.9; 15.7)	0.5 (0.3; 0.7)	** < 0.001**
Role functioning (10.53)	52.8 (30.7)	36.8 (31.9)	16.1 (9.9; 22.2)	0.5 (0.3; 0.7)	** < 0.001**
Emotional functioning (6.46)	75.9 (21.1)	74.0 (22.3)	1.9 (−2.1; 5.8)	0.1 (−0.1; 0.3)	0.353
Cognitive functioning (6.42)	79.8 (20.7)	74.5 (24.3)	5.3 (1.6; 9.0)	0.2 (0.1; 0.4)	0.005
Social functioning (7.78)	73.3 (22.7)	60.9 (31.3)	12.4 (6.5; 18.3)	0.5 (0.2; 0.7)	** < 0.001**
Fatigue (8.48)	48.6 (26.2)	57.5 (26.8)	−9.0 (−13.7; −4.2)	−0.3 (−0.5; −0.2)	** < 0.001**
Nausea and vomiting (4.95)	7.7 (14.6)	14.5 (21.4)	−6.8 (−11.8; −1.8)	−0.4 (−0.7; −0.1)	**0.008**
Pain (10.29)	32.9 (31.1)	38.1 (33.7)	−5.2 (−11.4; 1.1)	−0.2 (−0.4; 0.0)	0.105
Dyspnea (8.21)	28.9 (28.5)	34.2 (32.1)	−5.4 (−10.8; 0.0)	−0.2 (−0.4; 0.0)	0.052
Insomnia (9.30)	35.4 (32.7)	33.0 (29.8)	2.4 (−4.1; 8.9)	0.1 (−0.1; 0.3)	0.467
Appetite loss (8.73)	19.2 (27.4)	36.0 (35.4)	−16.8 (−24.4; −9.2)	−0.6 (−0.8; −0.3)	** < 0.001**
Constipation (8.37)	25.7 (29.5)	20.4 (27.6)	5.3 (−1.1; 11.7)	0.2 (−0.0; 0.4)	0.101
Diarrhea (6.54)	18.0 (28.2)	28.0 (33.0)	−10.0 (−17.3; −2.7)	−0.4 (−0.6; −0.1)	**0.008**
Financial difficulties (4.92)	12.4 (23.2)	12.2 (24.1)	0.2 (−3.7; 4.1)	0.0 (−0.2; 0.2)	0.924
*EORTC QLQ-MY20*	Q = 35*	Q = 34*			
Disease symptoms (6.77)	29.0 (24.5)	26.5 (20.1)	2.6 (−4.4; 9.5)	0.1 (−0.2; 0.4)	0.457
Side effect of treatment (4.32)	22.1 (16.1)	29.3 (14.0)	−7.2 (−11.6; −2.9)	−0.5 (−0.7; −0.2)	**0.002**
Future perspectives (8.16)	61.0 (24.8)	62.7 (23.8)	−1.8 (−7.4; 3.8)	−0.1 (−0.3; 0.2)	0.522
Body image (8.80)	72.4 (29.7)	67.6 (32.3)	4.7 (−7.7; 17.2)	0.2 (−0.3; 0.6)	0.445
*EORTC QLQ-CIPN20*	Q = 112	Q = 108			
Peripheral neuropathy (3.40)	15.0 (13.2)	19.6 (16.3)	−4.6 (−7.5; −1.7)	−0.3 (−0.5; −0.1)	**0.002**
*SF12v2*	Q = 33*	Q = 32*			
Physical Component Summary (3.41)	39.3 (11.5)	35.9 (9.1)	3.4 (−0.4; 7.2)	0.3 (−0.0; 0.7)	0.081
Mental Component Summary (3.49)	41.2 (10.7)	37.9 (10.6)	3.3 (−0.4; 7.0)	0.3 (−0.0; 0.6)	0.079

Salvage responses due to hospital admission, physical or mental reasons. P-values in bold are both statistically significant and clinically relevant

*Q* number of completed questionnaires, *EORTC QLQ-C30* European Organisation For Research And Treatment Of Cancer Quality Of Life Questionnaire, *EORTC QLQ-MY20* European Organisation For Research And Treatment Of Cancer Multiple Myeloma module, *EORTC QLQ-CIPN20* European Organisation For Research And Treatment Of Cancer Chemotherapy-Induced Peripheral Neuropathy, *SF12v2* Short-form health survey version 2–4-week recall, *SD* standard deviation. *The EORTC QLQ-MY20 and SF12v2 questionnaires were only included in the set of questionnaires for the patient to complete every 3 months. For EORTC QLQ-MY20, PRO completion rate for the Disease Symptoms domain is presented

For NR group 2 (technical difficulties, never received questionnaire or paper disappeared), the salvage mean scores were statically significant and clinically better in only 1 of 22 domains (future perspectives) compared to the previous on-time mean score before salvage response. For the NR group 3 (forgotten/overlooked questionnaires) and NR group 4 (other/no available reason), none of the salvage mean scores were statistically significant and clinically different compared to the previous on-time mean scores before salvage response. The mean scores, mean differences and p-values for NR group 2–4 are presented in Tables 4, 5, 6. In the analysis of all 802 salvage responses in total, we found that none of the salvage mean scores were statistically significant and clinically different compared to the previous on-time mean scores before salvage response; these are presented in the supplementary appendix Table 8S.

**Table 4:** Mean difference for non-responses group 2

Quality of life domains (minimal important difference threshold)	Mean previous on-time score before salvage response (SD)	Mean salvage score (SD) (Q = 110*)	Mean difference (95% confidence interval)	Standardized difference (95% confidence interval)	p-value
*EORTC QLQ-C30*	Q = 111	Q = 111			
Global quality of life (7.66)	58.1 (24.4)	60.4 (20.3)	−2.3 (−7.0; 2.4)	−0.1 (−0.3; 0.1)	0.325
Physical functioning (7.44)	70.7 (20.4)	68.8 (21.7)	1.9 (−0.9; 4.8)	0.1 (−0.0; 0.2)	0.186
Role functioning (10.53)	55.1 (29.8)	55.6 (29.0)	−0.5 (−5.2; 4.3)	−0.0 (−0.2; 0.2)	0.852
Emotional functioning (6.46)	79.0 (20.8)	80.1 (21.5)	−1.1 (−3.9; 1.7)	−0.1 (−0.2; 0.1)	0.434
Cognitive functioning (6.42)	78.1 (23.4)	78.2 (24.6)	−0.1 (−4.0; 3.8)	−0.0 (−0.2; 0.2)	0.958
Social functioning (7.78)	73.4 (25.1)	73.9 (26.7)	−0.5 (−3.7; 2.7)	−0.0 (−0.2; 0.1)	0.751
Fatigue (8.48)	44.2 (26.3)	41.8 (24.3)	2.4 (−2.1; 6.9)	0.1 (−0.1; 0.3)	0.292
Nausea and vomiting (4.95)	9.8 (19.1)	6.0 (13.1)	3.8 (0.2; 7.3)	0.2 (0.0; 0.5)	0.038
Pain (10.29)	35.7 (29.9)	34.1 (26.7)	1.7 (−3.6; 7.0)	0.1 (−0.1; 0.2)	0.537
Dyspnea (8.21)	22.6 (29.0)	25.5 (28.2)	−2.8 (−8.4; 2.7)	−0.1 (−0.3; 0.1)	0.316
Insomnia (9.30)	26.4 (28.1)	27.3 (31.2)	−0.9 (−6.5; 4.7)	−0.0 (−0.2; 0.2)	0.749
Appetite loss (8.73)	18.6 (26.8)	18.6 (26.8)	−0.0 (−6.2; 6.2)	−0.0 (−0.3; 0.3)	1.000
Constipation (8.37)	19.8 (30.3)	19.1 (25.7)	0.7 (−5.6; 7.0)	0.0 (−0.2; 0.3)	0.819
Diarrhea (6.54)	19.8 (27.5)	16.2 (22.5)	3.6 (−0.7; 7.9)	0.1 (−0.0; 0.3)	0.099
Financial difficulties (4.92)	8.6 (20.0)	7.6 (20.0)	1.0 (−2.3; 4.3)	0.1 (−0.1; 0.2)	0.552
*EORTC QLQ-MY20*	Q = 48*	Q = 46*			
Disease symptoms (6.77)	30.2 (19.6)	26.3 (16.7)	3.8 (−1.9; 9.5)	0.2 (−0.1; 0.5)	0.181
Side effect of treatment (4.32)	21.7 (14.7)	21.4 (15.6)	0.2 (−4.7; 5.2)	0.0 (−0.3; 0.4)	0.924
Future perspectives (8.16)	60.2 (21.7)	70.3 (19.9)	−10.1 (−16.3; −3.9)	−0.5 (−0.8; −0.2)	**0.002**
Body image (8.80)	63.9 (38.2)	73.9 (28.9)	−10.0 (−21.7; 1.7)	−0.3 (−0.7; 0.0)	0.091
*EORTC QLQ-CIPN20*	Q = 111	Q = 111			
Peripheral neuropathy (3.40)	18.1 (16.9)	19.5 (17.1)	−1.4 (−3.3; 0.6)	−0.1 (−0.2; 0.0)	0.169
*SF12v2*	Q = 44*	Q = 42*			
Physical Component Summary (3.41)	37.9 (10.8)	40.0 (10.3)	−2.1 (−5.6; 1.5)	−0.2 (−0.6; 0.1)	0.244
Mental Component Summary (3.49)	43.9 (10.2)	45.4 (10.6)	−1.5 (−4.7; 1.6)	−0.2 (−0.5; 0.2)	0.326

Salvage responses due to reason of technical difficulties, never received questionnaire or paper disappeared. P-values in bold are both statistically significant and clinically relevant

*Q* number of completed questionnaires, *EORTC QLQ-C30* European Organisation For Research And Treatment Of Cancer Quality Of Life Questionnaire, *EORTC QLQ-MY20* European Organisation For Research And Treatment Of Cancer Multiple Myeloma module, *EORTC QLQ-CIPN20* European Organisation For Research And Treatment Of Cancer Chemotherapy-Induced Peripheral Neuropathy, *SF12v2* Short-form health survey version 2–4-week recall, *SD* standard deviation. *The EORTC QLQ-MY20 and SF12v2 questionnaires were only included in the set of questionnaires for the patient to complete every 3 months. For EORTC QLQ-MY20, PRO completion rate for the Disease Symptoms domain is presented

**Table 5:** Mean differences non-responses group 3

Quality of life domains (minimal important difference threshold)	Mean previous on-time score before salvage response (SD)	Mean salvage score (SD)	Mean difference (95% confidence interval)	Standardized difference (95% confidence interval)	p-value
*EORTC QLQ-C30*	Q = 523	Q = 523			
Global quality of life (7.66)	59.2 (22.4)	58.9 (21.7)	0.3 (−2.3; 2.8)	0.0 (−0.1; 0.1)	0.844
Physical functioning (7.44)	70.1 (22.2)	69.9 (21.3)	0.2 (−2.6; 3.0)	0.0 (−0.1; 0.1)	0.892
Role functioning (10.53)	55.6 (30.5)	56.8 (29.7)	−1.2 (−4.4; 1.9)	−0.0 (−0.2; 0.1)	0.444
Emotional functioning (6.46)	78.6 (19.8)	78.1 (20.5)	0.4 (−1.2; 2.1)	0.0 (−0.1; 0.1)	0.621
Cognitive functioning (6.42)	79.4 (22.2)	78.6 (21.8)	0.8 (−1.1; 2.8)	0.0 (−0.1; 0.1)	0.390
Social functioning (7.78)	73.6 (26.6)	73.3 (27.5)	0.3 (−2.5; 3.0)	0.0 (−0.1; 0.1)	0.855
Fatigue (8.48)	43.7 (24.1)	43.3 (24.8)	0.4 (−2.0; 2.9)	0.0 (−0.1; 0.1)	0.728
Nausea and vomiting (4.95)	9.3 (16.3)	8.5 (15.7)	0.8 (−1.3; 2.8)	0.1 (−0.1; 0.2)	0.451
Pain (10.29)	31.7 (28.5)	30.1 (27.8)	1.6 (−1.5; 4.7)	0.1 (−0.1; 0.2)	0.308
Dyspnea (8.21)	25.8 (27.6)	23.5 (25.7)	2.3 (−0.7; 5.3)	0.1 (−0.0; 0.2)	0.133
Insomnia (9.30)	29.1 (29.2)	29.1 (30.6)	0.1 (−2.8; 3.0)	0.0 (−0.1; 0.1)	0.965
Appetite loss (8.73)	20.4 (27.2)	22.0 (28.8)	−1.6 (−4.7; 1.5)	−0.1 (−0.2; 0.1)	0.317
Constipation (8.37)	19.3 (26.0)	16.3 (24.6)	3.0 (−0.3; 6.3)	0.1 (−0.0; 0.3)	0.072
Diarrhea (6.54)	16.2 (24.6)	18.9 (26.2)	−2.7 (−5.6; 0.2)	−0.1 (−0.2; 0.0)	0.069
Financial difficulties (4.92)	10.0 (22.7)	11.3 (23.2)	−1.3 (−4.0; 1.4)	−0.1 (−0.2; 0.1)	0.353
*EORTC QLQ-MY20*	Q = 234*	Q = 229*			
Disease symptoms (6.77)	23.5 (20.1)	23.4 (18.9)	0.2 (−2.1; 2.4)	0.0 (−0.1; 0.1)	0.892
Side effect of treatment (4.32)	18.8 (16.0)	20.4 (15.8)	−1.6 (−3.7; 0.5)	−0.1 (−0.2; 0.0)	0.134
Future perspectives (8.16)	63.0 (27.0)	63.5 (26.9)	−0.5 (−3.8; 2.8)	−0.0 (−0.1; 0.1)	0.774
Body image (8.80)	73.4 (30.3)	70.5 (31.1)	3.0 (−1.0; 7.0)	0.1 (−0.0; 0.2)	0.141
*EORTC QLQ-CIPN20*	Q = 521	Q = 512			
Peripheral neuropathy (3.40)	14.8 (15.5)	16.6 (15.6)	−1.8 (−3.3; −0.3)	−0.1 (−0.2; −0.0)	0.021
*SF12v2*	Q = 218*	Q = 214*			
Physical Component Summary (3.41)	40.9 (9.8)	41.0 (10.0)	−0.1 (−1.6; 1.4)	−0.0 (−0.2; 0.1)	0.899
Mental Component Summary (3.49)	44.2 (12.3)	44.6 (11.1)	−0.4 (−2.2; 1.4)	−0.0 (−0.2; 0.1)	0.647

Salvage responses due to forgotten/overlooked the questionnaires. None of the p-values are both statistically significant and clinically relevant

*Q* number of completed questionnaires; *EORTC QLQ-C30* European Organisation For Research And Treatment Of Cancer Quality Of Life Questionnaire, *EORTC QLQ-MY20* European Organisation For Research And Treatment Of Cancer Multiple Myeloma module, *EORTC QLQ-CIPN20* European Organisation For Research And Treatment Of Cancer Chemotherapy-Induced Peripheral Neuropathy, *SF12v2* Short-form health survey version 2–4-week recall, *SD* standard deviation. *The EORTC QLQ-MY20 and SF12v2 questionnaires were only included in the set of questionnaires for the patient to complete every 3 months. For EORTC QLQ-MY20, PRO completion rate for the Disease Symptoms domain is presented

**Table 6:** Mean difference non-responses group 4

Quality of life domains (minimal important difference threshold)	Mean previous on-time score before salvage response (SD)	Mean salvage score (SD)	Mean difference (95% confidence interval)	Standardized difference (95% confidence interval)	p-value
*EORTC QLQ-C30*	Q = 55	Q = 55			
Global quality of life (7.66)	48.9 (20.1)	54.7 (21.6)	−5.8 (−13.4; 1.9)	−0.3 (−0.6; 0.1)	0.137
Physical functioning (7.44)	61.9 (20.3)	66.3 (20.9)	−4.5 (−11.3; 2.4)	−0.2 (−0.5; 0.1)	0.195
Role functioning (10.53)	41.5 (28.8)	51.2 (31.9)	−9.7 (−20.0; 0.6)	−0.3 (−0.7; 0.0)	0.064
Emotional functioning (6.46)	76.4 (18.8)	77.9 (22.1)	−1.5 (−8.6; 5.6)	−0.1 (−0.4; 0.3)	0.668
Cognitive functioning (6.42)	76.4 (21.0)	72.7 (23.9)	3.6 (−3.2; 10.4)	0.2 (−0.2; 0.5)	0.287
Social functioning (7.78)	67.0 (23.2)	73.9 (23.5)	−7.0 (−15.7; 1.8)	−0.3 (−0.7; 0.1)	0.115
Fatigue (8.48)	52.5 (24.0)	45.3 (22.1)	7.3 (−2.3; 16.8)	0.3 (−0.1; 0.8)	0.133
Nausea and vomiting (4.95)	13.3 (16.8)	10.3 (15.2)	3.0 (−2.6; 8.7)	0.2 (−0.2; 0.6)	0.285
Pain (10.29)	38.5 (25.0)	34.8 (27.6)	3.6 (−5.0; 12.3)	0.1 (−0.2; 0.5)	0.401
Dyspnea (8.21)	26.7 (25.2)	29.0 (32.4)	−2.3 (−13.9; 9.2)	−0.1 (−0.5; 0.3)	0.683
Insomnia (9.30)	26.1 (34.4)	21.2 (25.1)	4.8 (−6.3; 16.0)	0.2 (−0.2; 0.5)	0.384
Appetite loss (8.73)	25.5 (32.1)	20.0 (29.1)	5.5 (−4.0; 14.9)	0.2 (−0.1; 0.5)	0.250
Constipation (8.37)	21.2 (25.1)	17.6 (22.1)	3.6 (−2.9; 10.2)	0.2 (−0.1; 0.5)	0.267
Diarrhea (6.54)	14.5 (22.9)	17.0 (26.4)	−2.4 (−8.9; 4.0)	−0.1 (−0.4; 0.2)	0.450
Financial difficulties (4.92)	12.1 (23.5)	12.7 (25.2)	−0.6 (−5.5; 4.2)	−0.0 (−0.3; 0.2)	0.802
*EORTC QLQ-MY20*	Q = 23*	Q = 23*			
Disease symptoms (6.77)	17.7 (19.9)	21.6 (16.1)	−3.9 (−12.0; 4.3)	−0.2 (−0.8; 0.3)	0.337
Side effect of treatment (4.32)	24.1 (14.6)	23.4 (18.7)	0.7 (−5.0; 6.4)	0.0 (−0.3; 0.4)	0.795
Future perspectives (8.16)	64.1 (20.0)	69.1 (22.7)	−4.9 (−11.7; 1.8)	−0.2 (−0.6; 0.1)	0.144
Body image (8.80)	68.2 (28.1)	69.6 (30.0)	−1.4 (−15.9; 13.1)	−0.0 (−0.6; 0.5)	0.845
*EORTC QLQ-CIPN20*	Q = 55	Q = 54			
Peripheral neuropathy (3.40)	16.0 (13.3)	17.5 (16.0)	−1.5 (−4.0; 1.0)	−0.1 (−0.3; 0.1)	0.222
*SF12v2*	Q = 20*	Q = 20*			
Physical Component Summary (3.41)	38.3 (9.9)	37.0 (8.8)	1.3 (−4.1; 6.6)	0.1 (−0.5; 0.8)	0.634
Mental Component Summary (3.49)	41.4 (8.1)	45.2 (10.8)	−3.8 (−9.2; 1.6)	−0.4 (−1.0; 0.2)	0.161

Salvage responses due to other or no available reason for non-responses. None of the p-values are both statistically significant and clinically relevant

*Q* number of completed questionnaires; *EORTC QLQ-C30* European Organisation For Research And Treatment Of Cancer Quality Of Life Questionnaire, *EORTC QLQ-MY20* European Organisation For Research And Treatment Of Cancer Multiple Myeloma module, *EORTC QLQ-CIPN20* European Organisation For Research And Treatment Of Cancer Chemotherapy-Induced Peripheral Neuropathy, *SF12v2* Short-form health survey version 2–4-week recall, *SD* standard deviation; *The EORTC QLQ-MY20 and SF12v2 questionnaires were only included in the set of questionnaires for the patient to complete every 3 months. For EORTC QLQ-MY20, PRO completion rate for the Disease Symptoms domain is presented

As 28% of the NRs were never responses without salvage responses, we performed a post hoc analysis to investigate whether there were differences between mean previous on-time scores before salvage responses compared to mean previous on-time scores before never responses. For the NR group 1, four out of 22 domains had a statistically significant and clinically worse mean previous on-time score before never responses, compared to mean previous on-time score before salvage responses. Two domains (physical functioning and appetite loss) were the same as found in the main analysis suggesting that the patients providing a salvage response had reported a better HRQL score for those domains at the previously scheduled time point than the patients not providing a salvage response. The mean differences and p-values for NR group 1–4 and all NRs are presented in the supplementary appendix, Table S10–S14.

## Discussion

To our knowledge, this is the first study that explores the relationship between reasons for intermittent NR to scheduled PRO questionnaires and the missing data mechanism. We found that when questionnaires were not completed due to hospital admission or mental or physical reasons, a MNAR mechanism could be assumed for 10 out of 22 of the investigated domains. For the remaining domains, the results supported the MCAR mechanism. Further, our investigation revealed that intermittent NRs over the course of a longitudinal study had several reasons, and an individual patient may have NR due to more than one reason with different underlying missing data mechanisms. This knowledge has implication for reducing bias when analyzing and interpreting PRO data collected from any study including PRO data including clinical trials, surveys and cross-sectional studies.

Of the many approaches to handle NRs, the simplest is to use complete case analysis or replace the missing observation with a fixed value, such as the last value observed for that patient, or “last observation carried forward” (LOCF) [3]. An assumption for using LOCF is that the mean outcome remains constant [31]. In the current study, when we investigated the missing data mechanism for all intermittent NRs together and regardless of reason, the suggested missing data mechanism was the MCAR for all domains, which means LOCF would be an appropriate strategy for handling the intermittent NRs of our dataset as a whole. However, when we investigated the missing data mechanism based on reported reasons, nearly half of the domains in the group of intermittent NRs due to hospital admission or mental or physical reasons were suggested MNAR. Some authors advise against using the LOCF imputation strategy for two reasons [3, 31–33]; firstly, it reduces the natural variance in PRO scores at both the individual and sample levels, leading to underestimated standard errors and increased type 1 error rates. Secondly, it assumes the NRs are MCAR, and if this assumption is not met for all NRs, the results will be biased. Our findings offer another argument against using LOCF when the subset of intermittent missing data is due to hospital admission, or physical or mental reasons, as the missing data mechanism is likely to support the MNAR assumption, as was the case for, 12 and four domains respectively in our study.

Our study confirms that reasons for intermittent NRs can assist researchers and statisticians in speculating about the underlying missing data mechanism [3, 4, 7, 34–36]. Although the proportion of suggested MNAR data in our study was small (286/7534, 4%), the mean differences between previous on-time score and salvage score were large for some domains. The largest mean difference, 16.8 points, was found for appetite loss, which reflects the degree of potential bias. When considering the results of our post hoc analysis comparing the previous on-time score before NR for salvage responses with never responses for NR group 1 (hospital, mentally and physical), we found that patients not reporting salvage responses had reported even more appetite loss in the previous scheduled questionnaire. This suggests that the mean difference for appetite loss of 16.8 points might be underestimated. Again, this highlights the value of recording reasons for intermittent NRs for the study statistician to use the most appropriate statistical analyses method and for transparency for readers of subsequent trial findings.

We also found that the suggested missing data mechanisms were not uniform across all QoL domains within each NR group. In the group of intermittent NRs due to hospital admission, physical or mental reasons, several domains (emotional and cognitive functioning, pain, dyspnoea, insomnia, constipation, financial difficulties, disease symptoms, future perspectives, body image, and physical and mental component summary) were not significantly different from the previous on-time scores before salvage response. However, when considering the result of the post hoc analysis, the difference between mean previous on-time score before salvage response and salvage scores might be underestimated, as they are based only on NRs with salvage responses.

According to guidelines for including PROs clinical trial protocols, it is recommended that the statistical methods for handling missing PRO data should be stated in the PRO-specific components of a protocol [9, 10, 37]. In choosing appropriate statistical method for handling missing data, researchers should consider the expected PRO data completion rate, the expected missing data mechanism/s of the missing PRO data, and the proportion of MNAR missing data. In our analysis, we considered NR to each scheduled follow-up questionnaire separately by exploring missing data mechanism in NR groups based on reasons for NR, rather than exploring patient factors related to NR, as is typically done. In the mixed effects regression models, we corrected for dependence among observations from the same patient by clustered standard errors using the robust sandwich estimator. To consider NRs to each scheduled follow-up questionnaire separately differs from earlier published frameworks regarding treatment and reporting of missing data [8, 10, 38]. Often NRs in a study are considered homogeneously under the assumption they all have the same underlying missing data mechanism. We have challenged this assumption and found that 179/681 patients have NRs due to reasons in more than one of the four predefined NRs groups (data are presented in the supplementary appendix Table S5). This heterogeneity in reasons for NR is unknown at the time of writing a trial protocol, suggesting that a better strategy would be to include an investigation of missing data mechanism as part of the analysis, and based on the findings, choose an appropriate statistical method for handling NRs. In addition, our analyses were performed for each PRO domain. We found that not all mean salvage scores in NR group 1 were systematically different from the mean previous on-time score before salvage response, suggesting that the implications of NR can be domain-specific, and the impact of NR should be considered at a domain level.

This study has both strengths and limitations. The sample is large and utilize data from the QoL-MM study, where real-time monitoring and collection of reason for missed completion of scheduled questionnaires within a 7-day time window was included in the study design, which enabled 72% of assessments initially missed to be salvaged. Study nurse compliance in collecting and recording reasons for NRs was high (only 107/1108 (10%) missing). Further, we succeeded in implementing practical tracking and reminding procedures in a multicenter study to achieve a high level of information on reasons for NR and a high proportion of salvaged responses, which allowed us to address our methodological aim. We stated a priori expectations for the missing data mechanisms based on the logic behind the reasons for missingness, e.g. missing questionnaires due to hospital admission were expected to be MNAR and missing questionnaires due to technical difficulties were expected to be MCAR. Similar methodological studies in other patient groups and settings are required to see if our findings generalize to different types of patients. A limitation of the performed analyses is that they are based on salvage responses only, which were available for 72% of the non-responses. As a result of, our methods findings are based on observed PRO scores, all of which have a degree of inaccuracy, which is a clear limitation, but is the very nature of working with missing data. Because this study had an exploratory aim, we did not correct for multiple testing in the analyses performed, and our results should be interpreted with caution given the large number of tests performed and the variable sample size in Tables 3, 4, 5, 6. A further limitation is the inherent uncertainty in interpreting and aligning real-world reasons for missingness with statistical definitions of missing data mechanisms. Finally, we focused on intermittent missing data without considering monotone missing data, which is known to be predominantly MNAR in patients with advanced cancer [39].

## Conclusion

Based on observational data from patients with MM included in the longitudinal QoL-MM study, our findings suggest that intermittent non-responses due to hospital admissions and reasons related to physical and mental health may be “missing not at random” for about half of the PRO domains, while intermittent non-responses due to procedural errors, overlooked/forgetting to complete or other/no available reasons were likely to be “missing completely at random”. These findings emphasize the importance of recording reasons for intermittent non-responses as part of PRO data collection in clinical studies, as this information provides important guidance for statisticians about the missing data mechanisms in order to reduce the bias missing data might cause when analyzing datasets with intermittent non-responses and researchers with insights into potential biases when interpreting study findings. Our findings have implications for both clinical trials, surveys and cross-sectional studies.

## Supplementary Information

Below is the link to the electronic supplementary material.

Supplementary file1 (DOCX 107 KB)
