# Supplementary material for: Relationship between reasons for intermittent missing patient-reported outcomes data and missing data mechanisms
Source: Qual Life Res. 2024 Jun 16;33(9):2387–400. doi: 10.1007/s11136-024-03707-y (PMC11390842; doi:10.1007/s11136-024-03707-y)
Supplement: Supplementary file 1 — Supplementary file1 (DOCX 107 KB) [file 11136_2024_3707_MOESM1_ESM.docx]

**Supplementary appendix**

Article name: Relationship between reasons for intermittent missing patient-reported outcomes data and missing data mechanisms

Journal name: Quality of life Research

Table of Contents

[Study Procedures for real-time monitoring and ascertaining reasons for missing PRO data 2](#_Toc165441948)

[Demographic characteristics 4](#_Toc165441949)

[Table S1. Demographic characteristics at entry 4](#_Toc165441950)

[Reasons for intermittent non-responses 5](#_Toc165441951)

[Table S2. Reasons for intermittent non-responses, number of salvage and never responses 5](#_Toc165441952)

[Figure S1. Reasons for non-responses, percentage of salvage and never responses 6](#_Toc165441953)

[Table S3. Reasons for intermittent non-responses for patients completing follow-up questionnaires electronically 7](#_Toc165441954)

[Table S4. Reasons for intermittent non-responses for patients completing the follow-up questionnaires by paper 8](#_Toc165441955)

[Patients with multiple reasons for intermittent non-response 9](#_Toc165441956)

[Table S5. Number of patients with multiple reasons for non-responses 9](#_Toc165441957)

[Mean differences per non-response group 10](#_Toc165441958)

[Table S6. Mean differences non-responses group 1a (hospital admission). 10](#_Toc165441959)

[Table S7. Mean differences non-responses group 1b (physical and mental reasons). 11](#_Toc165441960)

[Table S8. Mean score differences for all salvage responses. 12](#_Toc165441961)

[Minimal important difference 13](#_Toc165441962)

[Table S9. Comparison of 0.3 standard deviation MID with published thresholds. 13](#_Toc165441963)

[Previous on-time score differences per group 14](#_Toc165441964)

[Table S10. Previous on-time score differences for non-responses group 1 (hospital admission, physical or mental reasons). 14](#_Toc165441965)

[Table S11. Previous on-time score difference non-responses group 2 (technical difficulties/never received/paper disappeared). 15](#_Toc165441966)

[Table S12. Previous on-time score difference non-responses group 3 (forgotten/overlooked). 16](#_Toc165441967)

[Table S13. Previous on-time score difference non-responses group 4 (other/no available reason). 17](#_Toc165441968)

[Table S14. Previous on-time score differences for all non-responses. 18](#_Toc165441969)

# **Study Procedures for real-time monitoring and ascertaining reasons for missing PRO data**

The following instructions were provided to study nurses to standardize procedures for real-time monitoring of PRO data completion and what to do in case non-response to a scheduled follow-up questionnaire was identified. Note that the pre-specified list of reasons for non-response is presented in Table 1 of the main paper.

If a patient has not completed any of the items of the EORTC QLQ-C30 or the EORTC QLQ-CIPN20 within seven days from the day for scheduled completion (target date), the questionnaire is defined as non-response, and the study office writes an email notification to the local study nurse. This procedure is being carried out by the study office every weekday during the entire study period. Within a 2-weekday timeframe, the local study nurse contacts the patient, ascertains the reason for non-response in dialogue with the patient and documents the main reason for non-response from a pre-specified list. In addition, the study nurse invites the patient to complete the questionnaire, which will then be denoted as a salvage response.

An equivalent procedure is being carried out for paper questionnaires. If a patient chooses to complete the questionnaires by paper, the local study nurse provides three sets of questionnaires to the patient together with a cover letter with instructions for completion, the study nurse´s name and telephone number. The local study nurse has typed the scheduled date of completion (target date) on the front of each set of questionnaires. The patients are instructed in completing the sets of questionnaires at the scheduled date and write the date for completion on the front of the questionnaires. The study nurse and the patient make an appointment for bringing the completed paper questionnaires to the hospital, and the date and time point are written in the cover letter provided to the patient. The patients are informed that if a paper questionnaire is not completed within the 7-day window, the study nurse will ask for the main reason for non-response, which the study nurse will document from a pre-specified list.

Paper questionnaires are uploaded as a document file to REDCap by the local study nurse and entered into REDCap by the study office. If a scheduled questionnaire from a patient using the paper completion method is not uploaded one month after the target date, the study office writes an email notification to the local study nurse, who investigates why the questionnaire has not been returned and uploaded. If the scheduled appointment for bringing the completed questionnaire to the hospital is missed, the local study nurse contacts the patient and determines what has happened. If the patient has not completed the scheduled questionnaire within the 7-day window, the study nurse ascertains the reason for non-response in dialogue with the patient and documents the main reason for non-response from a pre-specified list, presented in Table 1 of the manuscript.

When the three provided sets of questionnaires have been completed, it is the study nurses´ responsibility to provide the next three sets of questionnaires to the patient.

As the local study nurse or the study office are not automatically informed if an included patient had passed, death is included in the pre-specified list of intermittent non-responses. Non-response due to death is per definition a monotone non-response, and all reasons for non-response due to death were redefined as monotone non-responses at the time of data analysis.

If the study nurse chooses the category of “*Not possible to get in contact with the patient and find a reason*” the study nurses are instructed to meet the patient at the next hospital visit to ascertain the reason for non-response in dialogue with the patient and recode the reason for non-response in the REDCap database. This category was included in the pre-specified list for data management reasons, as when a reason for non-response is entered, the patient identification number is removed from the study office´s list of non-responses that require handling.

If the study nurse chooses the category of “*Other reason for not answering the questionnaire*”, an open box is made available, and the study nurse reports the reason for non-response in free text format. These reasons were recoded, if possible, by the first author before data analysis.

# **Demographic characteristics**

## **Table S1. Demographic characteristics at entry**

| **Characteristics** | **681 patients** |
| --- | --- |
| Mean age (SD) | 68.4 (9.2) |
| Median age, years (IQR) | 70.0 (60.0-75.0) |
| Age ≤ 65 years | 214 (31%) |
| Age 66-75 years | 295 (43%) |
| Age ≥ 76 years | 172 (25%) |
| Gender, female/male |  |
| Female | 277 (41%) |
| Male | 404 (59%) |
| Marital status |  |
| Married or cohabiting | 523 (77%) |
| Single^a^ | 158 (23%) |
| Charlson Comorbidity Index |  |
| 0 | 370 (54%) |
| 1 | 116 (17%) |
| 2 | 118 (17%) |
| ≥3 | 77 (11%) |
| Freiburg Comorbidity Index |  |
| 0 | 536 (79%) |
| 1 | 132 (19%) |
| 2 or 3 | 13 (2%) |
| IMWG myeloma frailty score |  |
| Fit | 362 (53%) |
| Intermediate Fitness | 208 (31%) |
| Frail | 111 (16%) |
| Karnofsky Performance Status Scale |  |
| 100% | 222 (33%) |
| 90% | 259 (38%) |
| 80% | 105 (15%) |
| ≥70% | 95 (14%) |
| Stages of disease |  |
| Newly diagnose | 403 (59%) |
| Relaps | 278 (41%) |
| Mode of questionnaire completion at entry |  |
| Electronic | 569 (84%) |
| Paper | 112 (16%) |

Abbreviations: SD, standard deviation; IQR, interquartile range; IMWG, International Myeloma Working Group.

^a^separated, divorced, widow or unmarried.

# **Reasons for intermittent non-responses**

## **Table S2. Reasons for intermittent non-responses, number of salvage and never responses**

|  | **Number of questionnaires** | **Number of times reason cited** | | |
| --- | --- | --- | --- | --- |
|  |  | **Total Number** | **Salvage responses** | **Never responses** |
| **Scheduled** | 7,534 | - | - | - |
| **On-time responses** | 6,426 | - | - | - |
| **Intermittent non-responses** | 1,108 | 1,108 | 802 | 306 |
| **Reasons for intermittent non-response** |  |  |  |  |
| 1. The patient is admitted to the Hematological Department | - | 82 | 44 | 38 |
| 2. The patient is admitted to another department than the Hematological Department | - | 47 | 13 | 34 |
| 3. The patient has overlooked/forgotten the questionnaire | - | 576 | 523 | 53 |
| 4. The patient had technical difficulties in answering the questionnaire | - | 44 | 41 | 3 |
| 5. The patient has never received the questionnaire (electronic or paper) | - | 81 | 68 | 13 |
| 6. The patient was not physically capable of answering the questionnaire | - | 61 | 26 | 35 |
| 7. The patient was not mentally capable of answering the questionnaire | - | 96 | 30 | 66 |
| 9. Not possible to get in contact with the patient and identify the reason | - | 34 | 20 | 14 |
| 10. Other or no available reason for non-response | - | 73 | 35 | 38 |
| 11. The paper questionnaire has disappeared and cannot be uploaded | - | 14 | 2 | 12 |

On-time response: patient completed at least one item of the EORTC QLQ-C30 or EORTC QLQ-CIPN20 questionnaires within the 7-day time window of a scheduled PRO assessment.

Non-response: patient did not complete any items of the EORTC QLQ-C30 or EORTC QLQ-CIPN20 questionnaires within seven days from the target day.

Salvage response: patient completed at least one item of the EORTC QLQ-C30 or EORTC QLQ-CIPN20 questionnaires, but later than seven days after the target day, following prompting by the study nurse.

Never responses: patient did not ever complete any items of the EORTC QLQ-C30 or EORTC QLQ-CIPN20 questionnaires.

Non-response due to death: Category 8 is excluded from the analysis as that category included non-responses due to death. Non-response due to death is per definition a monotone non-response, and all reasons for non-response due to death were redefined at the time of data analyses to monotone non-responses.

## **Figure S1. Reasons for non-responses, percentage of salvage and never responses**

## **Table S3. Reasons for intermittent non-responses for patients completing follow-up questionnaires electronically**

| **Electronic only** | **Number of question-naires** | **Number of times reason cited** | | |
| --- | --- | --- | --- | --- |
|  |  | **Total numbers** | **Salvage responses** | **Never responses** |
| **Scheduled** | 6,452 | - | - | - |
| **On-time responses** | 5,602 | - | - | - |
| **Reason for intermittent non-responses** | 850 | 850 | 655 | 195 |
| 1. The patient is admitted to the Hematological Department | - | 76 | 43 | 33 |
| 2. The patient is admitted to another department than the Hematological Department | - | 36 | 10 | 26 |
| 3. The patient has overlooked/forgotten the questionnaire | - | 463 | 440 | 23 |
| 4. The patient had technical difficulties in answering the questionnaire | - | 36 | 34 | 2 |
| 5. The patient has never received the questionnaire (electronic or paper) | - | 52 | 50 | 2 |
| 6. The patient was not physically capable of answering the questionnaire | - | 50 | 23 | 27 |
| 7. The patient was not mentally capable of answering the questionnaire | - | 73 | 24 | 49 |
| 9. Not possible to get in contact with the patient and identify the reason | - | 31 | 20 | 11 |
| 10. Other or no available reason for non-response | - | 31 | 11 | 20 |
| 11. The paper questionnaire has disappeared and can´t be uploaded^1^ | - | 2 | 0 | 2 |

On-time response: patient completed at least one item of the EORTC QLQ-C30 or EORTC QLQ-CIPN20 questionnaires within the 7-day time window of a scheduled PRO assessment.

Non-response: patient did not complete any items of the EORTC QLQ-C30 or EORTC QLQ-CIPN20 questionnaires within seven days from the target day.

Salvage response: patient completed at least one item of the EORTC QLQ-C30 or EORTC QLQ-CIPN20 questionnaires, but later than seven days after the target day, following prompting by the study nurse.

Never responses: patient did not ever complete any items of the EORTC QLQ-C30 or EORTC QLQ-CIPN20 questionnaires.

Non-response due to death: Category 8 is excluded from the analysis as that category included non-responses due to death. Non-response due to death is per definition a monotone non-response, and all reasons for non-response due to death were redefined at the time of data analyses to monotone non-responses.

^1^Category 11 was for disappeared paper questionnaires only. The table shows the patients using electronic completion at the time of analysis. Few patients changed the answering methods during the study, which explains why one of the electronic non-responses was due to disappeared paper questionnaire.

## **Table S4. Reasons for intermittent non-responses for patients completing the follow-up questionnaires by paper**

| **Paper only** | **Number of question-naires** | **Number of times reason cited** | | |
| --- | --- | --- | --- | --- |
|  |  | **Total numbers** | **Salvage responses** | **Never responses** |
| **Scheduled** | 1,082 | - | - | - |
| **On-time responses** | 824 | - | - | - |
| **Non-responses** | 258 | 258 | 147 | 111 |
| **Reason for intermittent non-responses** |  |  |  |  |
| 1. The patient wasadmitted to the Hematological Department | - | 6 | 1 | 5 |
| 2. The patient wasadmitted to another department than the Hematological Department | - | 11 | 3 | 8 |
| 3. The patient overlooked/forgot the questionnaire | - | 113 | 83 | 30 |
| 4. The patient had technical difficulties in answering the questionnaire | - | 8 | 7 | 1 |
| 5. The patient never received the questionnaire (electronic or paper) | - | 29 | 18 | 11 |
| 6. The patient was not physically capable of answering the questionnaire | - | 11 | 3 | 8 |
| 7. The patient was not mentally capable of answering the questionnaire | - | 23 | 6 | 17 |
| 9. Not possible to get in contact with the patient and identify the reason | - | 3 | 0 | 3 |
| 10. Other or no available reason for non-response | - | 42 | 24 | 18 |
| 11. The paper questionnaire has disappeared and can´t be uploaded | - | 12 | 2 | 10 |

On-time response: patient completed at least one item of the EORTC QLQ-C30 or EORTC QLQ-CIPN20 questionnaires within the 7-day time window of a scheduled PRO assessment.

Non-response: patient did not complete any items of the EORTC QLQ-C30 or EORTC QLQ-CIPN20 questionnaires within seven days from the target day.

Salvage response: patient completed at least one item of the EORTC QLQ-C30 or EORTC QLQ-CIPN20 questionnaires, but later than seven days after the target day, following prompting by the study nurse.

Never responses: patient did not ever complete any items of the EORTC QLQ-C30 or EORTC QLQ-CIPN20 questionnaires.

Non-response due to death: Category 8 is excluded from the analysis as that category included non-responses due to death. Non-response due to death is per definition a monotone non-response, and all reasons for non-response due to death were redefined at the time of data analyses to monotone non-responses.

# **Patients with multiple reasons for intermittent non-response**

## **Table S5. Number of patients with multiple reasons for non-responses**

|  | Number of patients  (N=681) |
| --- | --- |
| **Patients with complete data (on-time or salvage)** | 505 |
| **Patients with no non-responses (on-time responses only)** | 275 |
| **Patients with non-responses (salvage or never responses)** | 406 |
| **Non-response patient group 1** |  |
| Non-response patient group 1 only | 75 |
| Non-response patient group 1 and 2 | 16 |
| Non-response patient group 1 and 3 | 55 |
| Non-response patient group 1 and 4 | 12 |
| Non-response patient group 1, 2 and 3 | 17 |
| Non-response patient group 1, 2 and 4 | 1 |
| Non-response patient group 1, 3 and 4 | 15 |
| Non-response patient group 1, 2, 3 and 4 | 3 |
|  |  |
| **Non-response patient** **group 2** |  |
| Non-response patient group 2 only | 29 |
| Non-response patient group 2 and 3 | 29 |
| Non-response patient group 2 and 4 | 1 |
| Non-response patient group 2, 3 and 4 | 7 |
|  |  |
| **Non-response patient group 3** |  |
| Non-response patient group 3 only | 107 |
| Non-response patient group 3 and 4 | 23 |
|  |  |
| **Non-response patient** **group 4** |  |
| Non-response patient group 4 only | 16 |

Non-response patient group 1: Non-responses due to hospital admission, physical or mental reasons

Non-response patient group 2: Non-responses due to technical difficulties, never received questionnaire, or paper disappeared

Non-response patient group 3: Non-responses due to patients forgetting to complete or overlooking the questionnaires

Non-responses patient group 4: Non-responses due to other or no available reason for Non-responses

# **Mean differences per non-response group**

## **Table S6. Mean differences non-responses group 1a (hospital admission).**

P-values in **bold** are both statistically significant and clinically relevant.

| **Quality of life domains**  (minimal important difference threshold) | Mean previous on-time score before salvage response (SD) | Mean salvage score (SD) | Mean difference (95% Confidence interval) | p-value |
| --- | --- | --- | --- | --- |
| **EORTC QLQ-C30** | **Q=57** | **Q=57** |  |  |
| Global quality of life (7.66) | 51.3 (26.0) | 40.3 (24.2) | 11.0 (2.1; 19.9) | **0.017** |
| Physical functioning (7.44) | 64.4 (24.2) | 53.0 (22.2) | 11.4 (5.6; 17.3) | **<0.001** |
| Role functioning (10.53) | 48.2 (31.2) | 31.9 (29.9) | 16.3 (7.3; 25.4) | **0.001** |
| Emotional functioning (6.46) | 75.6 (20.2) | 71.5 (23.1) | 4.1 (-0.8; 9.0) | 0.097 |
| Cognitive functioning (6.42) | 81.0 (17.9) | 72.5 (23.9) | 8.5 (2.9; 14.1) | **0.004** |
| Social functioning (7.78) | 68.7 (21.8) | 57.0 (31.6) | 11.7 (4.4; 19.0) | **0.002** |
| Fatigue (8.48) | 52.0 (26.6) | 63.4 (23.6) | -11.4 (-17.4; -5.3) | **<0.001** |
| Nausea and vomiting (4.95) | 8.5 (14.5) | 19.3 (23.1) | -10.8 (-18.4; -3.3) | **0.006** |
| Pain (10.29) | 32.7 (32.7) | 41.2 (32.6) | -8.5 (-17.1; 0.2) | 0.055 |
| Dyspnea (8.21) | 39.3 (29.2) | 43.3 (33.3) | -4.0 (-12.5; 4.5) | 0.351 |
| Insomnia (9.30) | 38.1 (31.4) | 36.8 (31.3) | 1.3 (-8.0; 10.5) | 0.787 |
| Appetite loss (8.73) | 19.9 (27.4) | 40.9 (36.2) | -21.1 (-31.4; -10.7) | **<0.001** |
| Constipation (8.37) | 28.7 (31.1) | 19.3 (24.4) | 9.4 (0.1; 18.6) | **0.047** |
| Diarrhea (6.54) | 20.5 (28.0) | 32.2 (35.1) | -11.7 (-22.9; -0.5) | **0.041** |
| Financial difficulties (4.92) | 11.7 (22.3) | 14.0 (26.7) | -2.3 (-8.1; 3.5) | 0.422 |
| **EORTC QLQ-MY20** | **Q=17*** | **Q=16*** |  |  |
| Disease symptoms (6.77) | 32.7 (26.1) | 24.7 (15.8) | 8.0 (-3.4; 19.5) | 0.157 |
| Side effect of treatment (4.32) | 21.9 (15.4) | 32.4 (12.4) | -10.4 (-16.6; -4.2) | **0.003** |
| Future perspectives (8.16) | 58.2 (28.7) | 58.3 (28.3) | -0.2 (-10.9; 10.6) | 0.975 |
| Body image (8.80) | 76.5 (28.3) | 70.8 (34.2) | 5.6 (-12.8; 24.0) | 0.525 |
| **EORTC QLQ-CIPN20** | **Q=56** | **Q=54** |  |  |
| Peripheral neuropathy (3.40) | 19.0 (14.0) | 22.0 (16.2) | -3.0 (-6.9; 1.0) | 0.138 |
| **SF12v2** | **Q=15*** | **Q=15*** |  |  |
| Physical Component Summary (3.41) | 38.4 (12.5) | 34.5 (8.4) | 3.9 (-3.1; 11.0) | 0.255 |
| Mental Component Summary (3.49) | 43.7 (9.0) | 34.8 (9.2) | 8.9 (4.0; 13.9) | **0.001** |

Abbreviations: Q, number of completed questionnaires; EORTC QLQ-C30, European Organisation For Research And Treatment Of Cancer Quality Of Life Questionnaire; EORTC QLQ-MY20, European Organisation For Research And Treatment Of Cancer Multiple Myeloma module; EORTC QLQ-CIPN20, European Organisation For Research And Treatment Of Cancer Chemotherapy-Induced Peripheral Neuropathy; SF12v2, Short-form health survey version 2-4-week recall; SD, standard deviation.

*The EORTC QLQ-MY20 and SF12v2 questionnaires were only included in the set of questionnaires for the patient to complete every 3 months. For EORTC QLQ-MY20, PRO completion rate for the Disease Symptoms domain is presented.

## **Table S7. Mean differences non-responses group 1b (physical and mental reasons).**

P-values in **bold** are both statistically significant and clinically relevant.

| **Quality of life domains**  (minimal important difference threshold) | Mean previous on-time score before salvage response (SD) | Mean salvage score (SD) | Mean difference (95% Confidence interval) | p-value |
| --- | --- | --- | --- | --- |
| **EORTC QLQ-C30** | Q=56 | Q=56 |  |  |
| Global quality of life (7.66) | 56.1 (24.6) | 49.1 (23.8) | 7.0 (-1.1; 15.1) | 0.087 |
| Physical functioning (7.44) | 71.9 (21.1) | 60.7 (25.1) | 11.2 (4.2; 18.3) | **0.002** |
| Role functioning (10.53) | 57.4 (29.6) | 41.8 (33.3) | 15.6 (7.0; 24.2) | **0.001** |
| Emotional functioning (6.46) | 76.2 (22.1) | 76.6 (21.3) | -0.4 (-7.4; 6.6) | 0.909 |
| Cognitive functioning (6.42) | 78.6 (23.3) | 76.5 (24.8) | 2.1 (-3.9; 8.1) | 0.487 |
| Social functioning (7.78) | 78.0 (22.7) | 64.9 (30.8) | 13.1 (4.4; 21.7) | **0.004** |
| Fatigue (8.48) | 45.1 (25.6) | 51.5 (28.8) | -6.4 (-14.1; 1.4) | 0.105 |
| Nausea and vomiting (4.95) | 6.8 (14.8) | 9.5 (18.5) | -2.7 (-8.8; 3.4) | 0.381 |
| Pain (10.29) | 33.0 (29.7) | 34.8 (34.7) | -1.8 (-11.6; 8.0) | 0.716 |
| Dyspnea (8.21) | 18.5 (23.7) | 24.8 (28.1) | -6.4 (-12.9; 0.1) | 0.054 |
| Insomnia (9.30) | 32.7 (33.9) | 29.1 (28.0) | 3.6 (-5.7; 13.0) | 0.436 |
| Appetite loss (8.73) | 18.5 (27.6) | 31.0 (34.1) | -12.5 (-24.0; -1.0) | **0.033** |
| Constipation (8.37) | 22.6 (27.8) | 21.4 (30.8) | 1.2 (-8.5; 10.9) | 0.806 |
| Diarrhea (6.54) | 15.5 (28.4) | 23.6 (30.5) | -8.2 (-17.7; 1.3) | 0.090 |
| Financial difficulties (4.92) | 13.1 (24.4) | 10.3 (21.2) | 2.8 (-1.4; 7.0) | 0.188 |
| **EORTC QLQ-MY20** | Q=18* | Q=18* |  | |
| Disease symptoms (6.77) | 25.6 (23.1) | 28.1 (23.7) | -2.5 (-11.0; 6.1) | 0.551 |
| Side effect of treatment (4.32) | 22.2 (17.2) | 26.5 (15.1) | -4.4 (-10.9; 2.2) | 0.177 |
| Future perspectives (8.16) | 63.6 (20.8) | 66.7 (19.1) | -3.1 (-9.1; 2.9) | 0.295 |
| Body image (8.80) | 68.5 (31.3) | 64.8 (31.3) | 3.7 (-15.3; 22.7) | 0.687 |
| **EORTC QLQ-CIPN20** | Q=56 | Q=54 |  | |
| Peripheral neuropathy (3.40) | 11.0 (11.1) | 17.3 (16.3) | -6.2 (-10.6; -1.9) | **0.006** |
| **SF12v2** | Q=18* | Q=17* |  | |
| Physical Component Summary (3.41) | 40.0 (11.0) | 37.1 (9.7) | 2.9 (-1.8; 7.5) | 0.210 |
| Mental Component Summary (3.49) | 39.1 (11.7) | 40.7 (11.1) | -1.6 (-6.1; 2.9) | 0.470 |

Abbreviations: Q, number of completed questionnaires; EORTC QLQ-C30, European Organisation For Research And Treatment Of Cancer Quality Of Life Questionnaire; EORTC QLQ-MY20, European Organisation For Research And Treatment Of Cancer Multiple Myeloma module; EORTC QLQ-CIPN20, European Organisation For Research And Treatment Of Cancer Chemotherapy-Induced Peripheral Neuropathy; SF12v2, Short-form health survey version 2-4-week recall; SD, standard deviation.

*The EORTC QLQ-MY20 and SF12v2 questionnaires were only included in the set of questionnaires for the patient to complete every 3 months. For EORTC QLQ-MY20, PRO completion rate for the Disease Symptoms domain is presented.

## **Table S8. Mean score differences for all salvage responses.**

None of the mean differences in this table were both statistically significant and clinically relevant.

| **Quality of life domains**  (minimal important difference threshold) | Mean previous on-time score before salvage response (SD) | Mean salvage score (SD) | Mean difference (95% confidence interval) | Standardized difference (95% confidence interval) | p-value |
| --- | --- | --- | --- | --- | --- |
| **EORTC QLQ-C30** | Q=802 | Q=801 |  | | |
| Global quality of life (7.66) | 57.5 (23.1) | 56.8 (22.5) | 0.7 (-1.5; 3.0) | 0.0 (-0.1; 0.1) | 0.524 |
| Physical functioning (7.44) | 69.3 (22.0) | 67.6 (22.1) | 1.7 (-0.6; 4.0) | 0.1 (-0.0; 0.2) | 0.145 |
| Role functioning (10.53) | 54.2 (30.5) | 53.5 (30.8) | 0.7 (-2.1; 3.5) | 0.0 (-0.1; 0.1) | 0.612 |
| Emotional functioning (6.46) | 78.1 (20.0) | 77.8 (21.0) | 0.3 (-1.2; 1.7) | 0.0 (-0.1; 0.1) | 0.701 |
| Cognitive functioning (6.42) | 79.1 (22.1) | 77.5 (22.7) | 1.5 (-0.1; 3.2) | 0.1 (-0.0; 0.2) | 0.069 |
| Social functioning (7.78) | 73.1 (25.6) | 71.7 (28.0) | 1.4 (-1.0; 3.7) | 0.1 (-0.0; 0.1) | 0.257 |
| Fatigue (8.48) | 45.1 (24.8) | 45.2 (25.3) | -0.1 (-2.4; 2.1) | -0.0 (-0.1; 0.1) | 0.904 |
| Nausea and vomiting (4.95) | 9.4 (16.6) | 9.1 (16.4) | 0.3 (-1.6; 2.1) | 0.0 (-0.1; 0.1) | 0.769 |
| Pain (10.29) | 32.9 (28.9) | 32.1 (28.6) | 0.8 (-1.8; 3.4) | 0.0 (-0.1; 0.1) | 0.555 |
| Dyspnea (8.21) | 25.9 (27.7) | 25.6 (27.7) | 0.2 (-2.4; 2.8) | 0.0 (-0.1; 0.1) | 0.868 |
| Insomnia (9.30) | 29.4 (30.0) | 28.8 (30.3) | 0.6 (-1.9; 3.0) | 0.0 (-0.1; 0.1) | 0.641 |
| Appetite loss (8.73) | 20.3 (27.5) | 23.4 (30.0) | -3.0 (-6.0; -0.1) | -0.1 (-0.2; -0.0) | 0.041 |
| Constipation (8.37) | 20.4 (27.1) | 17.4 (25.0) | 3.1 (0.4; 5.8) | 0.1 (0.0; 0.2) | 0.026 |
| Diarrhea (6.54) | 16.9 (25.4) | 19.7 (27.0) | -2.8 (-5.2; -0.4) | -0.1 (-0.2; -0.0) | 0.022 |
| Financial difficulties (4.92) | 10.3 (22.5) | 11.0 (23.0) | -0.7 (-2.8; 1.4) | -0.0 (-0.1; 0.1) | 0.500 |
| **EORTC QLQ-MY20** | Q=340* | Q=332* |  | | |
| Disease symptoms (6.77) | 24.6 (20.7) | 24.0 (18.5) | 1.3 (-1.4; 3.9) | 0.0 (-0.1; 0.2) | 0.565 |
| Side effect of treatment (4.32) | 19.9 (15.8) | 21.7 (16.0) | -2.3 (-4.4; -0.2) | -0.1 (-0.2; 0.0) | 0.060 |
| Future perspectives (8.16) | 62.5 (25.6) | 64.7 (25.5) | -2.8 (-5.7; 0.1) | -0.1 (-0.2; 0.0) | 0.088 |
| Body image (8.80) | 71.6 (31.4) | 70.6 (30.7) | 2.1 (-2.3; 6.5) | 0.0 (-0.1; 0.2) | 0.594 |
| **EORTC QLQ-CIPN20** | Q=798 | Q=786 |  | | |
| Peripheral neuropathy (3.40) | 15.4 (15.3) | 17.5 (16.0) | -2.1 (-3.3; -0.9) | -0.1 (-0.2; -0.1) | 0.001 |
| **SF12v2** | Q=315* | Q=308* |  | | |
| Physical Component Summary (3.41) | 40.1 (10.2) | 40.0 (10.0) | 0.4 (-1.3; 2.0) | -0.1 (-0.2; -0.1) | 0.915 |
| Mental Component Summary (3.49) | 43.7 (11.7) | 44.1 (11.1) | -0.5 (-2.0; 1.0) | -0.0 (-0.2; 0.1) | 0.581 |

Abbreviations: Q, number of completed questionnaires; EORTC QLQ-C30, European Organisation For Research And Treatment Of Cancer Quality Of Life Questionnaire; EORTC QLQ-MY20, European Organisation For Research And Treatment Of Cancer Multiple Myeloma module; EORTC QLQ-CIPN20, European Organisation For Research And Treatment Of Cancer Chemotherapy-Induced Peripheral Neuropathy; SF12v2, Short-form health survey version 2-4-week recall; SD, standard deviation.

*The EORTC QLQ-MY20 and SF12v2 questionnaires were only included in the set of questionnaires for the patient to complete every 3 months. For EORTC QLQ-MY20, PRO completion rate for the Disease Symptoms domain is presented.

# **Minimal important difference**

## **Table S9. Comparison of 0.3 standard deviation MID with published thresholds.**

This table shows that our minimally important difference (MID) threshold of 0.3 SD, when expressed as a raw score based on published baseline standard deviations (SD), generally falls within the size range considered ‘small but clinically important’, which is very similar to the concept of minimally important difference. The exception is for the thresholds of EORTC QLQ-MY20 by Sully et al.^2^, which in comparison are larger than ours, especially for the domain of Side Effect of Treatment.

| **Quality of life domains** | Raw score MID estimated by 0.3 baseline mean score standard deviations | Size classification of 0.3SD MID according to the thresholds by Cocks et al.^1^ | MID thresholds developed by Sully et al.^2^ | Size classification of 0.3SD MID according to the thresholds by Kvam et al.^3^ |
| --- | --- | --- | --- | --- |
| **EORTC QLQ-C30** | |  |  |  |
| Global quality of life | 7.66 | Small |  | Small |
| Physical functioning | 7.44 | Small |  | Small |
| Role functioning | 10.53 | Small |  |  |
| Emotional functioning | 6.46 | - |  |  |
| Cognitive functioning | 6.42 | Small |  |  |
| Social functioning | 7.78 | Small |  |  |
| Fatigue | 8.48 | Small |  | Small |
| Nausea and vomiting | 4.95 | Small |  |  |
| Pain | 10.29 | Small |  | Small |
| Dyspnoea | 8.21 | Small |  |  |
| Insomnia | 9.30 | Small |  |  |
| Appetite loss | 8.73 | Small |  |  |
| Constipation | 8.37 | Small |  |  |
| Diarrhoea | 6.54 | Small |  |  |
| Financial difficulties | 4.92 | Small |  |  |
| **EORTC QLQ-MY20** | |  |  |  |
| Disease Symptoms | 6.77 |  | 10 |  |
| Side Effect of Treatment | 4.32 |  | 10 |  |
| Future Perspectives | 8.16 |  | 9 |  |
| Body Image | 8.80 |  | 13 |  |
| **EORTC QLQ-CIPN20** | |  |  |  |
| Peripheral neuropathy | 3.40 |  |  |  |
| **SF12v2** | |  |  |  |
| Physical Component Summery | 3.41 |  |  |  |
| Mental Component Summary | 3.49 |  |  |  |

References

^1^*Cocks K, King MT, Velikova G, Martyn St-James M, Fayers PM, Brown JM (2011) Evidence-based guidelines for determination of sample size and interpretation of the European Organisation for the Research and Treatment of Cancer Quality of Life Questionnaire Core 30. J Clin Oncol 29 (1):89-96*

*^2^Sully K, Trigg A, Bonner N, Moreno‐Koehler A, Trennery C, Shah N, Yucel E, Panjabi S, Cocks K. Estimation of minimally important differences and responder definitions for EORTC QLQ‐MY20 scores in multiple myeloma patients. European journal of haematology. 2019 Nov;103(5):500-9.*

*^3^Kvam AK, Wisloff F, Fayers PM (2010) Minimal important differences and response shift in health-related quality of life; a longitudinal study in patients with multiple myeloma. Health and quality of life outcomes 8:79.*

# **Previous on-time score differences per group**

**Table S10. Previous on-time score differences for non-responses group 1 (hospital admission, physical or mental reasons).**

Mean previous on-time score before salvage responses compared to mean previous on-time score before never responses for non-responses due to hospital admission, physical or mental reasons. P-values in **bold** are both statistically significant and clinically relevant.

| **Quality of life domains**  (minimal important difference threshold) | Mean previous on-time score before salvage response (SD) | Mean previous on-time score before never response (SD) | Mean difference (95% Confidence interval) | p-value |
| --- | --- | --- | --- | --- |
| **EORTC QLQ-C30** | Q=113 | Q=171 |  | |
| Global quality of life (7.66) | 53.7 (25.3) | 48.5 (25.3) | -5.2 (-12.2; 1.8) | 0.142 |
| Physical functioning (7.44) | 68.1 (22.9) | 58.4 (25.2) | -9.8 (-16.4; -3.1) | **0.004** |
| Role functioning (10.53) | 52.8 (30.7) | 44.4 (33.8) | -8.4 (-17.3; 0.4) | 0.062 |
| Emotional functioning (6.46) | 75.9 (21.1) | 72.5 (21.3) | -3.4 (-9.2; 2.5) | 0.257 |
| Cognitive functioning (6.42) | 79.8 (20.7) | 73.7 (25.8) | -6.1 (-12.1; -0.1) | 0.046 |
| Social functioning (7.78) | 73.3 (22.7) | 68.1 (29.6) | -5.2 (-12.7; 2.3) | 0.173 |
| Fatigue (8.48) | 48.6 (26.2) | 53.0 (27.0) | 4.4 (-2.6; 11.5) | 0.216 |
| Nausea and vomiting (4.95) | 7.7 (14.6) | 12.5 (18.9) | 4.9 (0.4; 9.3) | 0.033 |
| Pain (10.29) | 32.9 (31.1) | 45.5 (34.3) | 12.6 (3.3; 21.9) | **0.008** |
| Dyspnea (8.21) | 28.9 (28.5) | 24.7 (31.1) | -4.2 (-11.9; 3.5) | 0.282 |
| Insomnia (9.30) | 35.4 (32.7) | 29.9 (29.7) | -5.6 (-13.4; 2.3) | 0.166 |
| Appetite loss (8.73) | 19.2 (27.4) | 31.6 (29.7) | 12.4 (4.9; 20.0) | **0.001** |
| Constipation (8.37) | 25.7 (29.5) | 24.9 (32.6) | -0.8 (-9.5; 7.9) | 0.855 |
| Diarrhea (6.54) | 18.0 (28.2) | 17.1 (26.1) | -0.8 (-8.6; 6.9) | 0.829 |
| Financial difficulties (4.92) | 12.4 (23.2) | 9.4 (19.5) | -2.9 (-9.4; 3.6) | 0.372 |
| **EORTC QLQ-MY20** | Q=35* | Q=55* |  | |
| Disease symptoms (6.77) | 29.0 (24.5) | 39.2 (27.6) | 10.1 (-1.2; 21.5) | 0.079 |
| Side effect of treatment (4.32) | 22.1 (16.1) | 26.5 (17.7) | 4.4 (-2.8; 11.6) | 0.230 |
| Future perspectives (8.16) | 61.0 (24.8) | 54.9 (27.7) | -6.0 (-17.3; 5.3) | 0.295 |
| Body image (8.80) | 72.4 (29.7) | 66.7 (33.6) | -5.7 (-18.8; 7.4) | 0.388 |
| **EORTC QLQ-CIPN20** | Q=112 | Q=168 |  | |
| Peripheral neuropathy (3.40) | 15.0 (13.2) | 14.3 (14.3) | -0.7 (-4.5; 3.1) | 0.711 |
| **SF12v2** | Q=33* | Q=52* |  | |
| Physical Component Summary (3.41) | 39.3 (11.5) | 34.1 (10.6) | -5.1 (-10.1; -0.2) | **0.042** |
| Mental Component Summary (3.49) | 41.2 (10.7) | 38.6 (13.3) | -2.7 (-8.0; 2.6) | 0.319 |

Abbreviations: Q, number of completed questionnaires; EORTC QLQ-C30, European Organisation For Research And Treatment Of Cancer Quality Of Life Questionnaire; EORTC QLQ-MY20, European Organisation For Research And Treatment Of Cancer Multiple Myeloma module; EORTC QLQ-CIPN20, European Organisation For Research And Treatment Of Cancer Chemotherapy-Induced Peripheral Neuropathy; SF12v2, Short-form health survey version 2-4-week recall; SD, standard deviation.

*The EORTC QLQ-MY20 and SF12v2 questionnaires were only included in the set of questionnaires for the patient to complete every 3 months. For EORTC QLQ-MY20, PRO completion rate for the Disease Symptoms domain is presented.

**Table S11. Previous on-time score difference non-responses group 2 (technical difficulties/never received/paper disappeared).**

Mean previous on-time score before salvage response compared to mean previous on-time score before never response for non-responses due to technical difficulties, never received questionnaire or paper disappeared. P-values in **bold** are both statistically significant and clinically relevant.

| **Quality of life domains**  (minimal important difference threshold) | Mean previous on-time score before salvage response (SD) | Mean previous on-time score before never response (SD) | Mean difference (95% confidence interval) | p-value |
| --- | --- | --- | --- | --- |
| **EORTC QLQ-C30** | Q=111 | Q=28 |  | |
| Global quality of life (7.66) | 58.1 (24.4) | 56.0 (23.1) | -2.2 (-11.9; 7.6) | 0.662 |
| Physical functioning (7.44) | 70.7 (20.4) | 68.5 (27.6) | -2.3 (-14.3; 9.7) | 0.708 |
| Role functioning (10.53) | 55.1 (29.8) | 58.9 (32.9) | 3.8 (-11.3; 18.9) | 0.617 |
| Emotional functioning (6.46) | 79.0 (20.8) | 81.0 (24.6) | 2.0 (-8.2; 12.2) | 0.702 |
| Cognitive functioning (6.42) | 78.1 (23.4) | 73.8 (30.6) | -4.3 (-16.1; 7.6) | 0.476 |
| Social functioning (7.78) | 73.4 (25.1) | 81.0 (26.7) | 7.5 (-3.8; 18.9) | 0.190 |
| Fatigue (8.48) | 44.2 (26.3) | 40.5 (32.6) | -3.7 (-18.0; 10.6) | 0.607 |
| Nausea and vomiting (4.95) | 9.8 (19.1) | 7.7 (20.5) | -2.0 (-10.2; 6.2) | 0.625 |
| Pain (10.29) | 35.7 (29.9) | 30.4 (34.6) | -5.4 (-21.9; 11.1) | 0.520 |
| Dyspnea (8.21) | 22.6 (29.0) | 28.6 (29.7) | 5.9 (-8.0; 19.9) | 0.401 |
| Insomnia (9.30) | 26.4 (28.1) | 35.7 (31.3) | 9.3 (-5.1; 23.7) | 0.204 |
| Appetite loss (8.73) | 18.6 (26.8) | 19.0 (27.9) | 0.4 (-12.5; 13.4) | 0.948 |
| Constipation (8.37) | 19.8 (30.3) | 29.8 (30.5) | 9.9 (-3.9; 23.8) | 0.157 |
| Diarrhea (6.54) | 19.8 (27.5) | 21.4 (27.5) | 1.6 (-12.6; 15.8) | 0.822 |
| Financial difficulties (4.92) | 8.6 (20.0) | 9.5 (22.0) | 1.0 (-8.2; 10.1) | 0.835 |
| **EORTC QLQ-MY20** | Q=48* | Q=9* |  | |
| Disease symptoms (6.77) | 30.2 (19.6) | 19.1 (16.0) | -11.0 (-23.0; 1.0) | 0.071 |
| Side effect of treatment (4.32) | 21.7 (14.7) | 12.8 (12.7) | -8.9 (-18.2; 0.4) | 0.060 |
| Future perspectives (8.16) | 60.2 (21.7) | 62.5 (20.5) | 2.3 (-12.1; 16.8) | 0.749 |
| Body image (8.80) | 63.9 (38.2) | 83.3 (35.6) | 19.4 (-7.7; 46.6) | 0.156 |
| **EORTC QLQ-CIPN20** | Q=111 | Q=28 |  | |
| Peripheral neuropathy (3.40) | 18.1 (16.9) | 14.2 (12.0) | -4.0 (-10.2; 2.3) | 0.215 |
| **SF12v2** | Q=44* | Q=9* |  | |
| Physical Component Summary (3.41) | 37.9 (10.8) | 39.2 (13.8) | 1.3 (-8.4; 10.9) | 0.791 |
| Mental Component Summary (3.49) | 43.9 (10.2) | 51.3 (7.6) | 7.4 (1.4; 13.4) | **0.017** |

Abbreviations: Q, number of completed questionnaires; EORTC QLQ-C30, European Organisation For Research And Treatment Of Cancer Quality Of Life Questionnaire; EORTC QLQ-MY20, European Organisation For Research And Treatment Of Cancer Multiple Myeloma module; EORTC QLQ-CIPN20, European Organisation For Research And Treatment Of Cancer Chemotherapy-Induced Peripheral Neuropathy; SF12v2, Short-form health survey version 2-4-week recall; SD, standard deviation.

*The EORTC QLQ-MY20 and SF12v2 questionnaires were only included in the set of questionnaires for the patient to complete every 3 months. For EORTC QLQ-MY20, PRO completion rate for the Disease Symptoms domain is presented.

## **Table S12. Previous on-time score difference non-responses group 3 (forgotten/overlooked).**

Mean previous on-time score before salvage response compared to mean previous on-time score before never response for non-responses due to forgotten/overlooked the questionnaires. The p-value in **bold** is both statistically significant and clinically relevant.

| **Quality of life domains**  (minimal important difference threshold) | Mean previous on-time score before salvage response (SD) | Mean previous on-time score before never response (SD) | Mean difference (95% confidence interval) | p-value |
| --- | --- | --- | --- | --- |
| **EORTC QLQ-C30** | Q=523 | Q=53 |  | |
| Global quality of life (7.66) | 59.2 (22.4) | 48.7 (20.5) | -10.4 (-17.4; -3.5) | **0.003** |
| Physical functioning (7.44) | 70.1 (22.2) | 64.3 (17.6) | -5.8 (-11.6; 0.1) | 0.053 |
| Role functioning (10.53) | 55.6 (30.5) | 49.7 (27.4) | -5.9 (-15.2; 3.3) | 0.209 |
| Emotional functioning (6.46) | 78.6 (19.8) | 77.0 (19.7) | -1.5 (-7.9; 4.9) | 0.642 |
| Cognitive functioning (6.42) | 79.4 (22.2) | 78.9 (24.9) | -0.5 (-9.0; 8.0) | 0.911 |
| Social functioning (7.78) | 73.6 (26.6) | 69.8 (28.7) | -3.8 (-13.4; 5.8) | 0.440 |
| Fatigue (8.48) | 43.7 (24.1) | 51.4 (24.1) | 7.6 (-0.5; 15.8) | 0.067 |
| Nausea and vomiting (4.95) | 9.3 (16.3) | 8.5 (14.1) | -0.8 (-5.7; 4.1) | 0.757 |
| Pain (10.29) | 31.7 (28.5) | 33.0 (28.8) | 1.3 (-8.3; 11.0) | 0.784 |
| Dyspnea (8.21) | 25.8 (27.6) | 25.2 (28.4) | -0.7 (-10.1; 8.8) | 0.891 |
| Insomnia (9.30) | 29.1 (29.2) | 28.3 (28.0) | -0.8 (-10.9; 9.3) | 0.872 |
| Appetite loss (8.73) | 20.4 (27.2) | 29.6 (37.9) | 9.2 (-3.3; 21.6) | 0.149 |
| Constipation (8.37) | 19.3 (26.0) | 15.7 (21.3) | -3.6 (-10.7; 3.4) | 0.312 |
| Diarrhea (6.54) | 16.2 (24.6) | 16.4 (25.0) | 0.1 (-8.3; 8.5) | 0.975 |
| Financial difficulties (4.92) | 10.0 (22.7) | 12.8 (25.7) | 2.8 (-4.9; 10.6) | 0.475 |
| **EORTC QLQ-MY20** | Q=234* | Q=16* |  | |
| Disease symptoms (6.77) | 23.5 (20.1) | 26.2 (21.6) | 2.7 (-7.9; 13.4) | 0.615 |
| Side effect of treatment (4.32) | 18.8 (16.0) | 23.0 (15.9) | 4.2 (-3.7; 12.1) | 0.292 |
| Future perspectives (8.16) | 63.0 (27.0) | 62.5 (17.6) | -0.5 (-10.0; 9.0) | 0.916 |
| Body image (8.80) | 73.4 (30.3) | 60.4 (34.9) | -13.0 (-30.0; 3.9) | 0.130 |
| **EORTC QLQ-CIPN20** | Q=521 | Q=53 |  | |
| Peripheral neuropathy (3.40) | 14.8 (15.5) | 14.7 (17.3) | -0.1 (-6.4; 6.2) | 0.978 |
| **SF12v2** | Q=218* | Q=16* |  | |
| Physical Component Summary (3.41) | 40.9 (9.8) | 38.3 (7.9) | -2.6 (-6.6; 1.5) | 0.213 |
| Mental Component Summary (3.49) | 44.2 (12.3) | 38.0 (12.9) | -6.2 (-12.5; 0.1) | 0.055 |

Abbreviations: Q, number of completed questionnaires; EORTC QLQ-C30, European Organisation For Research And Treatment Of Cancer Quality Of Life Questionnaire; EORTC QLQ-MY20, European Organisation For Research And Treatment Of Cancer Multiple Myeloma module; EORTC QLQ-CIPN20, European Organisation For Research And Treatment Of Cancer Chemotherapy-Induced Peripheral Neuropathy; SF12v2, Short-form health survey version 2-4-week recall; SD, standard deviation.

*The EORTC QLQ-MY20 and SF12v2 questionnaires were only included in the set of questionnaires for the patient to complete every 3 months. For EORTC QLQ-MY20, PRO completion rate for the Disease Symptoms domain is presented.

## **Table S13. Previous on-time score difference non-responses group 4 (other/no available reason).**

Mean previous on-time score before salvage response compared to mean previous on-time score before never response for non-responses due to other or no available reason for non-responses. None of the results are both statistically significant and clinically relevant.

| **Quality of life domains**  (minimal important difference threshold) | Mean previous on-time score before salvage response (SD) | Mean previous on-time score before never response (SD) | Mean difference (95% confidence interval) | p-value |
| --- | --- | --- | --- | --- |
| **EORTC QLQ-C30** | Q=55 | Q=52 |  | |
| Global quality of life (7.66) | 48.9 (20.1) | 44.6 (26.7) | -4.3 (-17.3; 8.7) | 0.509 |
| Physical functioning (7.44) | 61.9 (20.3) | 58.3 (31.5) | -3.6 (-20.9; 13.7) | 0.681 |
| Role functioning (10.53) | 41.5 (28.8) | 42.3 (36.7) | 0.8 (-17.4; 19.0) | 0.931 |
| Emotional functioning (6.46) | 76.4 (18.8) | 72.1 (20.8) | -4.3 (-16.6; 8.0) | 0.488 |
| Cognitive functioning (6.42) | 76.4 (21.0) | 72.1 (28.7) | -4.2 (-19.5; 11.0) | 0.580 |
| Social functioning (7.78) | 67.0 (23.2) | 69.6 (26.2) | 2.6 (-8.7; 14.0) | 0.645 |
| Fatigue (8.48) | 52.5 (24.0) | 57.1 (30.6) | 4.5 (-12.1; 21.1) | 0.588 |
| Nausea and vomiting (4.95) | 13.3 (16.8) | 13.1 (17.6) | -0.2 (-9.6; 9.2) | 0.968 |
| Pain (10.29) | 38.5 (25.0) | 47.4 (35.0) | 9.0 (-7.5; 25.4) | 0.282 |
| Dyspnea (8.21) | 26.7 (25.2) | 31.4 (30.1) | 4.7 (-10.2; 19.6) | 0.530 |
| Insomnia (9.30) | 26.1 (34.4) | 44.2 (36.0) | 18.2 (-3.2; 39.5) | 0.094 |
| Appetite loss (8.73) | 25.5 (32.1) | 26.9 (33.0) | 1.5 (-13.6; 16.5) | 0.847 |
| Constipation (8.37) | 21.2 (25.1) | 14.7 (25.9) | -6.5 (-17.9; 5.0) | 0.264 |
| Diarrhea (6.54) | 14.5 (22.9) | 28.2 (37.6) | 13.7 (-8.2; 35.5) | 0.217 |
| Financial difficulties (4.92) | 12.1 (23.5) | 7.2 (19.2) | -4.9 (-16.3; 6.5) | 0.392 |
| **EORTC QLQ-MY20** | Q=23* | Q=20* |  | |
| Disease symptoms (6.77) | 17.7 (19.9) | 36.4 (22.4) | 18.7 (2.3; 35.0) | 0.027 |
| Side effect of treatment (4.32) | 24.1 (14.6) | 30.2 (20.5) | 6.1 (-10.0; 22.3) | 0.447 |
| Future perspectives (8.16) | 64.1 (20.0) | 56.1 (34.3) | -8.0 (-35.1; 19.1) | 0.551 |
| Body image (8.80) | 68.2 (28.1) | 58.3 (37.3) | -9.8 (-33.4; 13.7) | 0.401 |
| **EORTC QLQ-CIPN20** | Q=54 | Q=52 |  | |
| Peripheral neuropathy (3.40) | 16.0 (13.3) | 23.7 (23.1) | 7.7 (-5.3; 20.8) | 0.243 |
| **SF12v2** | Q=20* | Q=18* |  | |
| Physical Component Summary (3.41) | 38.3 (9.9) | 34.9 (9.4) | -3.4 (-11.1; 4.3) | 0.377 |
| Mental Component Summary (3.49) | 41.4 (8.1) | 40.2 (12.3) | -1.2 (-9.3; 7.0) | 0.774 |

Abbreviations: Q, number of completed questionnaires; EORTC QLQ-C30, European Organisation For Research And Treatment Of Cancer Quality Of Life Questionnaire; EORTC QLQ-MY20, European Organisation For Research And Treatment Of Cancer Multiple Myeloma module; EORTC QLQ-CIPN20, European Organisation For Research And Treatment Of Cancer Chemotherapy-Induced Peripheral Neuropathy; SF12v2, Short-form health survey version 2-4-week recall; SD, standard deviation.

*The EORTC QLQ-MY20 and SF12v2 questionnaires were only included in the set of questionnaires for the patient to complete every 3 months. For EORTC QLQ-MY20, PRO completion rate for the Disease Symptoms domain is presented.

## **Table S14. Previous on-time score differences for all non-responses.**

Mean previous on-time score before salvage response compared to mean previous on-time score before never response for non-responses for all non-responses. P-values in **bold** are both statistical significant and clinical relevant.

| **Quality of life domains**  (minimal important difference threshold) | Mean previous on-time score before salvage response (SD) | Mean previous on-time score before never response (SD) | Mean difference (95% Confidence interval) | p-value |
| --- | --- | --- | --- | --- |
| **EORTC QLQ-C30** | Q=802 | Q=304 |  | |
| Global quality of life (7.66) | 57.5 (23.1) | 48.6 (24.7) | -9.0 (-13.2; -4.7) | **<0.001** |
| Physical functioning (7.44) | 69.3 (22.0) | 60.3 (25.6) | -9.0 (-13.8; -4.2) | **<0.001** |
| Role functioning (10.53) | 54.2 (30.5) | 46.3 (33.4) | -7.9 (-13.9; -1.9) | 0.010 |
| Emotional functioning (6.46) | 78.1 (20.0) | 74.0 (21.4) | -4.1 (-7.9; -0.2) | 0.038 |
| Cognitive functioning (6.42) | 79.1 (22.1) | 74.3 (26.6) | -4.7 (-9.5; 0.1) | 0.053 |
| Social functioning (7.78) | 73.1 (25.6) | 69.8 (28.7) | -3.2 (-8.2; 1.7) | 0.199 |
| Fatigue (8.48) | 45.1 (24.8) | 52.3 (27.9) | 7.2 (2.2; 12.2) | 0.005 |
| Nausea and vomiting (4.95) | 9.4 (16.6) | 11.5 (18.1) | 2.1 (-0.9; 5.1) | 0.168 |
| Pain (10.29) | 32.9 (28.9) | 42.3 (34.0) | 9.4 (3.4; 15.3) | 0.002 |
| Dyspnea (8.21) | 25.9 (27.7) | 26.2 (30.3) | 0.4 (-4.9; 5.7) | 0.893 |
| Insomnia (9.30) | 29.4 (30.0) | 32.6 (31.1) | 3.1 (-2.6; 8.9) | 0.284 |
| Appetite loss (8.73) | 20.3 (27.5) | 29.3 (31.7) | 9.0 (3.7; 14.2) | **0.001** |
| Constipation (8.37) | 20.4 (27.1) | 22.0 (30.0) | 1.6 (-3.7; 6.8) | 0.555 |
| Diarrhea (6.54) | 16.9 (25.4) | 19.3 (28.5) | 2.4 (-3.2; 8.0) | 0.393 |
| Financial difficulties (4.92) | 10.3 (22.5) | 9.6 (20.8) | -0.6 (-4.2; 3.0) | 0.726 |
| **EORTC QLQ-MY20** | Q=340* | Q=100* |  | |
| Disease symptoms (6.77) | 24.6 (20.7) | 34.8 (25.5) | 10.1 (3.7; 16.6) | **0.002** |
| Side effect of treatment (4.32) | 19.9 (15.8) | 25.4 (18.0) | 5.5 (0.6; 10.4) | **0.029** |
| Future perspectives (8.16) | 62.5 (25.6) | 57.0 (27.2) | -5.5 (-12.7; 1.8) | 0.138 |
| Body image (8.80) | 71.6 (31.4) | 65.3 (34.8) | -6.3 (-14.9; 2.3) | 0.148 |
| **EORTC QLQ-CIPN20** | Q=798 | Q=301 |  | |
| Peripheral neuropathy (3.40) | 15.4 (15.3) | 16.0 (16.8) | 0.6 (-3.1; 4.2) | 0.749 |
| **SF12v2** | Q=315* | Q=95* |  | |
| Physical Component Summary (3.41) | 40.1 (10.2) | 35.5 (10.3) | -4.7 (-7.4; -1.9) | **0.001** |
| Mental Component Summary (3.49) | 43.7 (11.7) | 40.0 (13.0) | -3.7 (-6.9; -0.5) | **0.023** |

Abbreviations: Q, number of completed questionnaires; EORTC QLQ-C30, European Organisation For Research And Treatment Of Cancer Quality Of Life Questionnaire; EORTC QLQ-MY20, European Organisation For Research And Treatment Of Cancer Multiple Myeloma module; EORTC QLQ-CIPN20, European Organisation For Research And Treatment Of Cancer Chemotherapy-Induced Peripheral Neuropathy; SF12v2, Short-form health survey version 2-4-week recall; SD, standard deviation.

*The EORTC QLQ-MY20 and SF12v2 questionnaires were only included in the set of questionnaires for the patient to complete every 3 months. For EORTC QLQ-MY20, PRO completion rate for the Disease Symptoms domain is presented.
